# Inferring Domain-Domain Interactions from Protein-Protein Interactions with Formal Concept Analysis

**DOI:** 10.1371/journal.pone.0088943

**Published:** 2014-02-19

**Authors:** Susan Khor

**Affiliations:** Department of Computer Science, Memorial University of Newfoundland, St John’s, Newfoundland and Labrador, Canada; University of South Florida College of Medicine, United States of America

## Abstract

Identifying reliable domain-domain interactions will increase our ability to predict novel protein-protein interactions, to unravel interactions in protein complexes, and thus gain more information about the function and behavior of genes. One of the challenges of identifying reliable domain-domain interactions is domain promiscuity. Promiscuous domains are domains that can occur in many domain architectures and are therefore found in many proteins. This becomes a problem for a method where the score of a domain-pair is the ratio between observed and expected frequencies because the protein-protein interaction network is sparse. As such, many protein-pairs will be non-interacting and domain-pairs with promiscuous domains will be penalized. This domain promiscuity challenge to the problem of inferring reliable domain-domain interactions from protein-protein interactions has been recognized, and a number of work-arounds have been proposed. This paper reports on an application of Formal Concept Analysis to this problem. It is found that the relationship between formal concepts provides a natural way for rare domains to elevate the rank of promiscuous domain-pairs and enrich highly ranked domain-pairs with reliable domain-domain interactions. This piggybacking of promiscuous domain-pairs onto less promiscuous domain-pairs is possible only with concept lattices whose attribute-labels are not reduced and is enhanced by the presence of proteins that comprise both promiscuous and rare domains.

## Introduction

Proteins comprise domains which are evolutionary conserved sequence segments with the ability to fold and be functional. An important class of domains mediates protein-protein interactions (PPIs); although not all interactions between proteins can be attributed to interactions between domains, and not all domains in multi-domain proteins play a direct role in protein interaction. Nonetheless, many computational methods which seek to predict PPIs with high accuracy rely on computationally inferred domain-domain interactions (DDIs), e.g. [1].

Ideally, the inferred DDIs used to support the predicted PPIs are highly reliable themselves, that is there is a large overlap between the set of inferred DDIs and the set of DDIs confirmed to interact by 3D crystal structures of proteins. This latter set is referred to in this paper as the gold standard domain-domain interactions (GDDIs). This ideal is desirable not only to tease out specific interactions in a protein complex, but also to give predictive power to protein-protein interaction prediction methods (more on this point later in this section).

However, using GDDIs to predict PPIs generates a large number of false positives (non-interacting protein-pairs predicted as interacting) and thus reduces the accuracy of the prediction method. The large number of false positives stem from the fact that GDDIs are enriched with *promiscuous domains*. Promiscuous domains can occur in many domain architectures [2] and thus appear in many proteins. But since the PPI network is sparse, many of these protein-pairs will be non-interacting.

It is parsimonious to re-use domain-pairs that can interact to facilitate PPIs. Indeed, many DDIs are conserved across organisms by evolution [3] and there is a high degree of DDI re-use by PPIs [4]. In theory, PPI prediction methods which depend on inferred DDIs rely on the presence of this parsimony. The basic underlying thinking is DDIs inferred from PPIs in the training set can then be used to predict PPIs in the test set. Fundamental to the success of this strategy is a commonality between the proteins in the training and test sets, at least in the form of domain-pairs. When this commonality is reduced, e.g. through the use of rare DDIs to predict PPIs, the power (ability to generalize from sample to population) of a prediction method weakens. This flaw in existing computational PPI prediction methods was demonstrated in [5] wherein the predictive performances of seven PPI prediction methods deteriorated significantly as the intersection between the training protein set and the test protein set decreased to null.

The ‘drift towards rare domain-pairs’ phenomenon in PPI prediction methods has been noted [6]. Such rare domain-pairs comprise domains which occur infrequently in a given protein sample but occurs in interacting protein-pairs so that rare domain-pairs appear to be highly reliable DDIs and good indicators of putative PPIs (since they dampen the increase in false positives). However, rare domain-pairs are often *not* GDDIs. Promiscuous domains are observed to be heavily involved in PPI mediation [2]. Further, rare domain-pairs have weak predictive value since by their nature, they are not commonly found in proteins and therefore the information that they interact is less reusable for the purpose of predicting PPIs. I suggest that the ‘drift towards rare domain-pairs’ phenomenon is partly a consequence of how computational PPI prediction methods are evaluated. However, the ‘drift towards rare domain-pairs’ is also because *promiscuity prevents GDDIs from being highly ranked* in computational methods to infer DDIs. Recognizing this domain promiscuity problem, additional measures have been taken to counteract its effects when inferring DDIs from PPIs, e.g. [6]–[8].

In this paper, I propose that Formal Concept Analysis (FCA) [9] is a feasible way to overcome the promiscuity problem for detecting GDDIs from a given set of PPIs. The proposed concept-based scoring method is a more discrete approach than previous methods, and I believe the first use of FCA in this manner. The success of the concept-based scoring method lies with the *piggy-backing* potential of promiscuous domain-pairs. Piggy-backing occurs when a domain-pair improves its score because either one or both of its domain partners happen to occupy the same attribute-label set as one or more rarer domains. Piggy-backing potential is possible only in concept lattices that are not attribute-reduced, and is enhanced by the presence of many mixed architecture proteins. One of the challenges of doing bioinformatics research is the volatility and variety of bioinformatics datasets which exerts a high validation cost for any new method. Therefore, it is useful to understand why a bioinformatics method works and under what conditions. Identifying such conditions also automatically suggests the limits of a method. Towards this end, effort is made to investigate conditions favourable to the proposed concept-based domain-pair ranking method.

## Materials

### Basic Definitions

Let *P* be the set of proteins and *D* the set of domains. Every protein in P comprises one or more domains in *D*. D(*x*) denotes the finite set of domains for protein *x*. If | D(*x*) | = 1, *x* is a single-domain protein; if | D(*x*) | > 1, *x* is a multi-domain protein. The set of proteins which contains domain *a* is P(*a*) = { *x* ∈ *P* | *a* ∈ D(*x*) }. The frequency of domain *a* in *P* is N(*a*) = | P(*a*) |. *x* = D(*x*) = {*a*, *b*, *c*} where *x* ∈ *P* and {*a*, *b*, *c*} ⊂ *D* denotes protein *x* is its set of domains D(*x*) which in turn comprises domains *a*, *b* and *c*.

The set of PPIs is a relation on *P*. This relation is symmetric, i.e. if (*x, y*) is an interacting protein-pair, then so is (*y*, *x*). It is possible for proteins to self-interact. Let (*x*, *y*)^1^ denote (*x*, *y*) ∈ the set of PPIs. (*x*, *y*)^1^ implies proteins *x* and *y* come from the same organism, i.e.: *O*(*x*) = *O*(*y*). The set of non-PPIs is also a symmetric relation on 3, and a non-interacting protein-pair (*x*, *y*)^0^ also implies *O*(*x*) = *O*(*y*). *O*(*x*) = *O*(*y*) implies either (*x*, *y*)^1^ or (*x*, *y*)^0^. There may be pairs in *P* × *P* which are neither interacting nor non-interacting because they do not satisfy the same organism condition.

The set of DDIs is a symmetric relation on *D*, and domain self-interaction is possible. A protein-pair (*x*, *y*) generates domain-pairs, each of which may or may not be reliable, through the cross-product of their domains, i.e. D(*x*) × D(*y*). A domain-pair (*a*, *b*) generates a set of protein-pairs, each of which may or may not be interacting, through the cross-product of their respective protein sets, i.e. P(*a*) × P(*b*). For a domain-pair (*a*, *b*) to be a DDI, it must generate at least one interacting protein-pair.

### The Riley Dataset and its Characteristics

**Figure 1:**
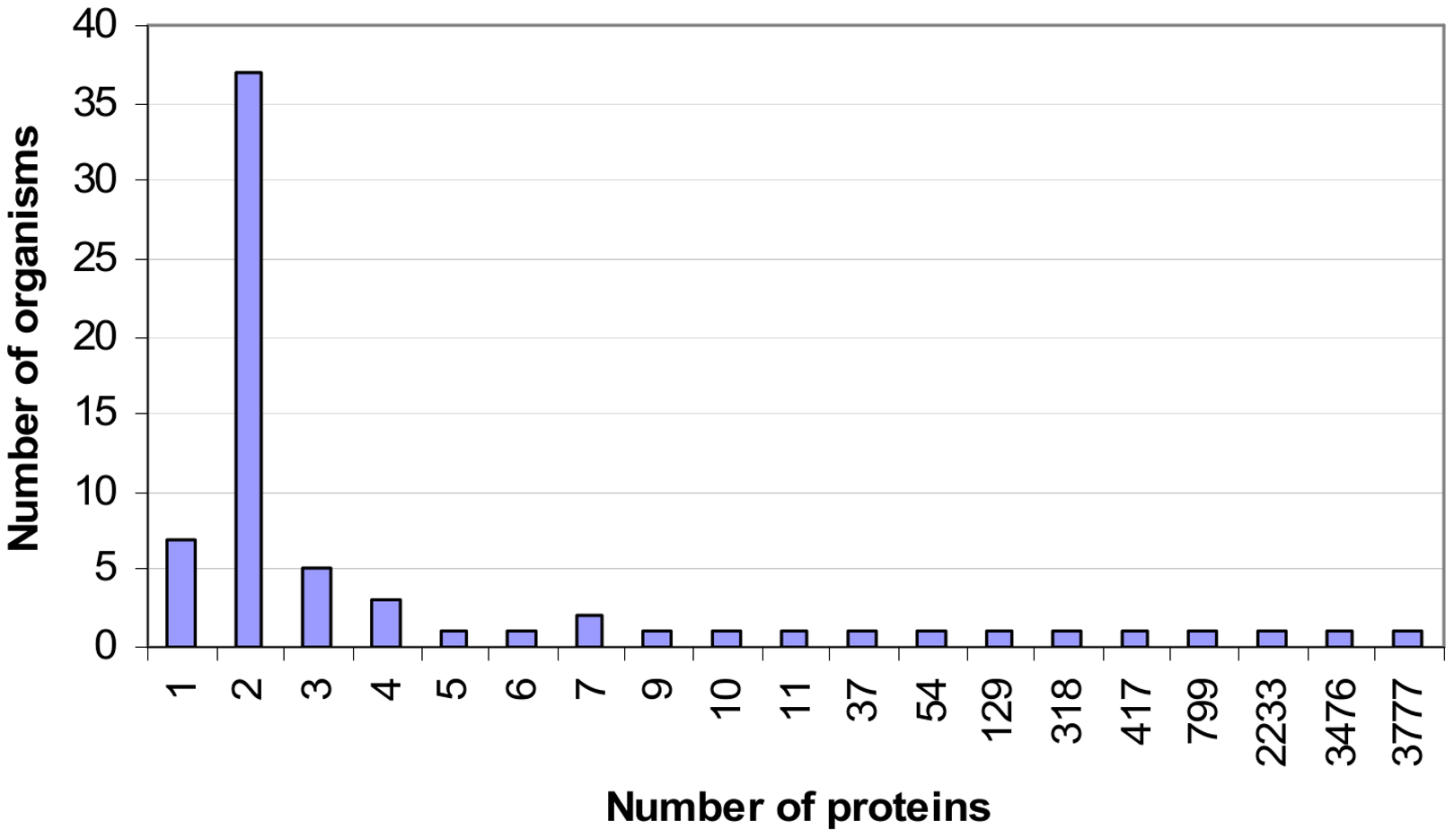
Breakdown of proteins by organism in the Riley dataset [8]. More than half of the organisms have only two proteins each. The top four organisms with the largest number of proteins are Fruitfly, Yeast, Worm and Human.

The Riley dataset [8] has been re-used in a number of studies, e.g. [6], [10]. This dataset comprises 11,403 proteins from 68 organisms. Figure 1 gives the breakdown of proteins by organism. The organisms with the four largest numbers of proteins in the Riley dataset are Fruitfly, Yeast, Worm and Human.

The proteins are associated with 12,455 Pfam-A and Pfam-B domains. Amongst the set of proteins are 26,032 protein-protein interactions (PPIs). Interactions and non-interactions between protein-pairs are restricted to proteins from the same organism [8]. The interaction of two proteins *x* and *y* implies interactions between the domains of *x* and the domains of *y*. The Riley set of proteins, domains and PPIs generate 177,233 putative domain-domain interactions (DDIs). Amongst these possible DDIs are 783 gold standard domain-domain interactions (GDDIs). These GDDIs were obtained from [6], and are domain-pairs whose interaction has been confirmed in *i*Pfam [16] from PDB crystal structures. Over half (403/783 = 51.57%) of the GDDIs are self-interacting (homotypic), but less than 1% (1262/176450) of the non-GDDIs are self-interacting. DDIs which mediate PPIs are enriched with homotypic domain-pairs [3], [11]. A PPI with at least one GDDI is a gold-PPI (GPPI). There are 2,326 GPPIs in the Riley dataset, 546 of which have a single domain-pair. Table 1 gives a sample of organisms found in the Riley dataset with their respective protein, domain and gold domain sizes. A gold domain is a domain that is involved in at least one GDDI.

**Table 1:** A sample of organisms in the Riley dataset with their respective protein, domain and gold domain sizes.

Organism	Number of proteins	Number of unique domains	Number of gold domains
All	11,403	12,455	642
*Drosophila melanogaster*	3,777	5,454	387
*Saccharomyces cerevisiae*	3,476	4,949	392
*Caenorhabditis elegans*	2,233	3,746	289
*Homo sapiens*	799	1,816	209
*Schizosaccharomyces pombe*	10	15	7
*Bacillus subtilis*	9	10	3

A gold domain is a domain that is involved in at least one GDDI. These GDDIs were obtained from [6], and are domain-pairs whose interaction has been confirmed in *i*Pfam [16] from PDB crystal structures.

In the following sub-sections, the Riley dataset is examined to support assertions made in this paper. Specifically, the data characteristics of interest are:

- Highly reliable domain-domain interactions (GDDIs) are enriched with promiscuous domains.
- GDDIs generate significantly more true positive PPIs and also more false positive PPIs than non-GDDIS. More true positive PPIs agrees with the parsimony or the re-use principle for GDDIs, and more false positive PPIs accords with the promiscuity of gold domains.
- Protein domain architectures are mostly a mix of rare and promiscuous domains.

#### GDDIs are significantly enriched with promiscuous domains

**Figure 2:**
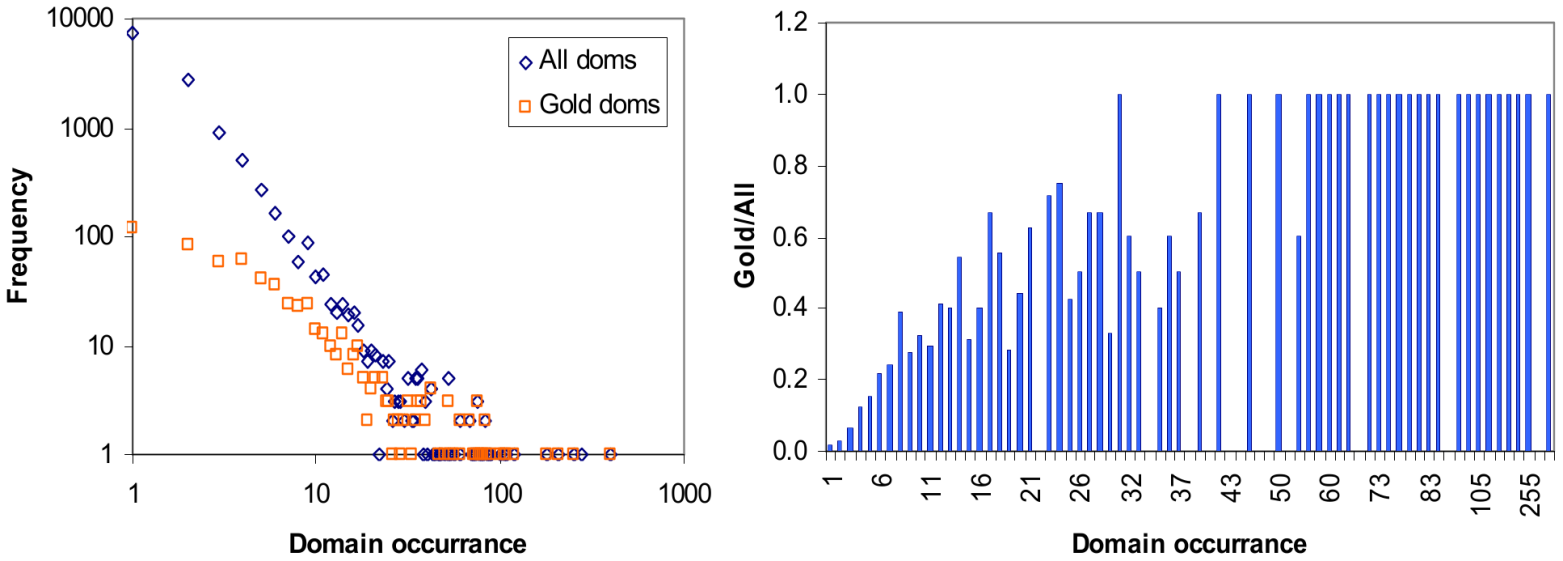
Domains do not occur with the same frequency. There are rare and promiscuous domains (left). Rare domains are domains which appear infrequently in the set of proteins. The rare domains outnumber the promiscuous domains several fold. The rare domains occur less frequently in the set of gold domains than in the set of all domains. In general, the more frequently a domain occurs, the more likely it is to be a gold domain (right). The same patterns are observed when proteins are confined to single organisms (Figure S1 in File S1).

All domains that participate in at least one GDDI were pooled and their frequencies were compared against the frequency of occurrence of all 12,455 domains. In agreement with previous observation in [4], few domains occur much more frequently and most domains occur infrequently. The log-log plot in Figure 2 shows the right-skewed distribution of domain occurrence which is exhibited more clearly by the set of all domains than the set of gold domains (even though the set of all domains is much larger than the set of gold domains). The difference in frequency distributions is significant. Analysis with R’s Wilcox.test confirms that the set of gold domains is significantly more promiscuous than the set of all domains. Hence, GDDIs are significantly enriched with promiscuous domains. A *gold domain* is a domain that participates in at least one gold standard domain-domain interaction (GDDI). There are 642 gold domains in the Riley dataset. A domain is more promiscuous if it occurs more frequently in a given set of proteins, i.e. given *P* and {a, b} ⊂ *D*, N(*a*)>N(*b*) implies *a* is more promiscuous than *b*.

The bar chart in Figure 2 gives the ratio of gold domains against all domains for every domain occurrence value. Gold/All = *y* for a domain occurrence value of *x* means *y* fraction of all domains that occurs *x* times are gold domains. The tendency is for Gold/All to equal 1 as domain occurrence increases. This supports the notion that GDDIs are significantly enriched with promiscuous domains. This conclusion is not surprising given that the GDDIs are sourced from the iPfam database [6] and a significant positive correlation between domain promiscuity and the number of structural interactions in iPfam was observed in [2].

Because the dataset comprises multi-organisms, there is a possibility that the heavy imbalance between rare and promiscuous domains in the set of all domains may be due to the many organisms with only a handful of proteins (Figure 1) in the dataset. To address this concern, the analysis is repeated on the set of domains specific to the four organisms with the most proteins in the Riley dataset, i.e. Fruitfly, Yeast, Worm and Human (Table 1). The results (Figure S1 in File S1) exhibit the same pattern as in Figure 2 and the differences between corresponding frequency distributions are significant. Thus the larger frequency of rare domains in the set of all domains is not an artifact of the multi-organism dataset. This does not mean that all gold domains are promiscuous or that none of the non-gold domains are promiscuous. Also for organisms with very few proteins, the sample size is not large enough to produce a significant difference, e.g. *S. pombe*.

The previous test examined the promiscuity of single domains. In the following test, the promiscuity of domain-pairs are examined by quantifying the promiscuity of a domain pair (*a*, *b*) as [N(*a*)+N(*b*)]/2. The promiscuity of GDDIs is then compared against the promiscuity of all non-GDDIs. R’s Wilcox.test confirms that GDDIs have significantly larger promiscuity scores than non-GDDIs.

#### GDDIs generate significantly more true positive and more false positive PPIs than non-GDDIs

The set of all interacting protein-pairs (PPIs) and the set of all non-interacting protein-pairs (non-PPIs) were generated for each of the 177,233 putative domain-domain interactions (DDIs). A domain-pair (*a*, *b*) generates a set of protein-pairs, each of which may or may not be interacting, through the cross-product of their protein sets, i.e. P(*a*) × P(*b*). A DDI is either a GDDI or a non-GDDI. The set of DDIs comprise 783 GDDIs and 176,450 non-GDDIs. A PPI prediction for a protein-pair (*x*, *y*) predicts that protein *x* interacts with protein *y*. This prediction is a true positive if (*x*, *y*)^1^ can be found in the given set of PPIs and a false positive otherwise.

R’s t.test (non-homogeneous variance) and Wilcox.test were used to compare the sizes of the PPI sets generated by GDDIs and by non-GDDIs, and the sizes of the non-PPIs sets produced by GDDIs and by non-GDDIs. The statistical tests confirm that GDDIs generate significantly larger sets of PPIs than non-GDDIs and that GDDIs generate significantly larger sets of non-PPIs than non-GDDIs. Thus, GDDIs generate significantly more true positive and more false positive PPIs than non-GDDIs. More true positive PPIs agrees with the parsimony or the re-use principle for GDDIs, and more false positive PPIs accords with the promiscuity of gold domains.

#### Protein domain architectures are mostly a mixture of rare and promiscuous domains

**Figure 3:**
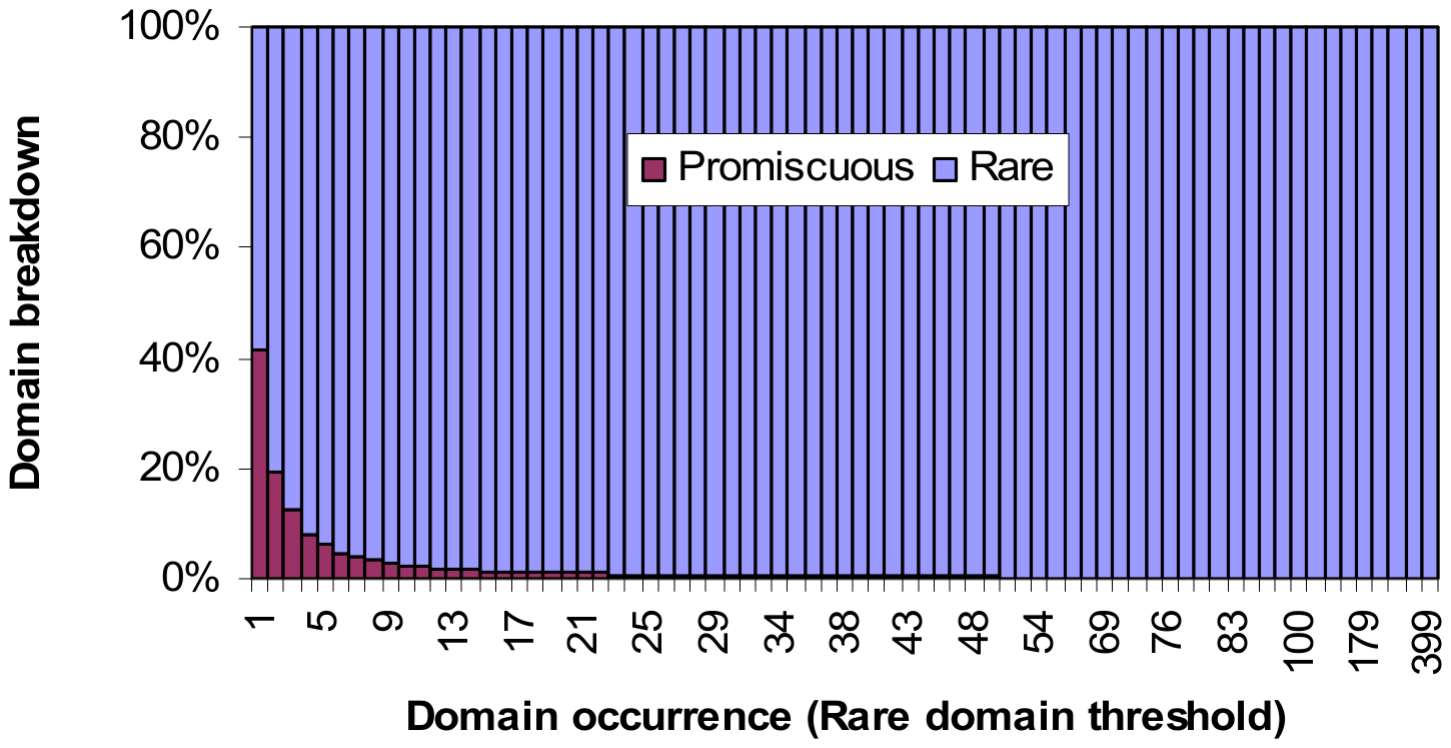
Ratio of promiscuous to rare domains. At rare domain threshold *x*, domains which occur at most *x* times in a protein set, i.e. N(*d*) ≤ *x*, are classified as rare. When the rare domain threshold is 1, 58.5% of all domains are rare, and the remaining 41.5% are promiscuous, i.e. occurs 2 or more times in the set of proteins. When the rare domain threshold is 2, 80.4% of all domains are rare. The percentage of domains that are promiscuous drops to 6.1% when the rare domain threshold is 5.

Promiscuous domains are those which occur frequently in proteins. Rare domains are those which occur infrequently in proteins. The ratio of promiscuous to rare domains varies with the rare domain threshold (Figure 3). At *rare domain threshold x*, domains which occur ≤ *x* times in the protein set are classified as rare. While a rare domain may occur in only a handful of proteins, collectively the set of rare domains occur in many proteins. When the rare domain threshold is 2, 54.8% of the proteins in the Riley dataset have at least one rare domain.

**Figure 4:**
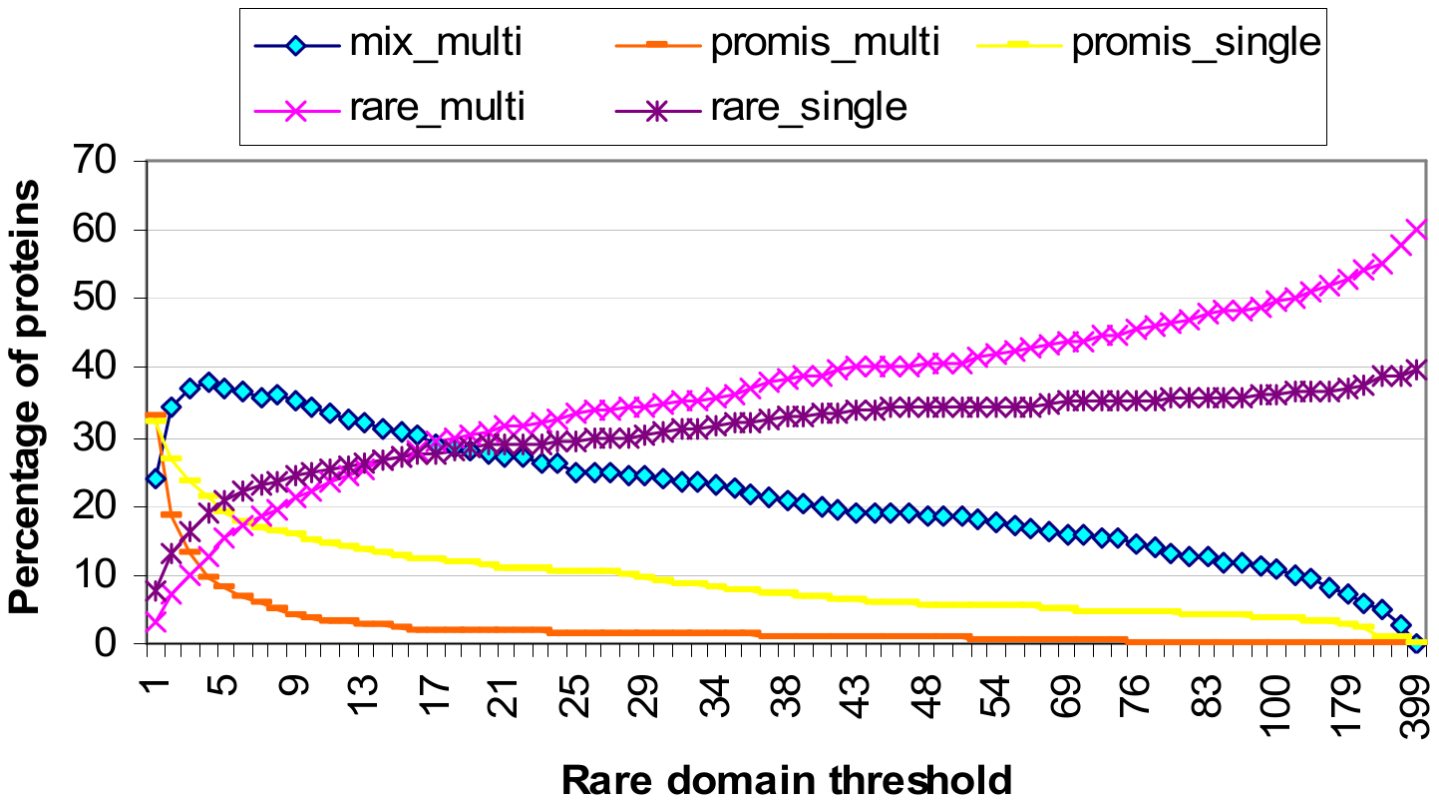
Breakdown of proteins by architecture (domain composition). As more domains are classified rare, the proportion of proteins comprising only-rare domains increases, while the proportion of proteins comprising only-promiscuous domains decreases. Mixed architecture proteins comprise both rare and promiscuous domains. They make up the largest percentage (at least a third) of the protein population when the rare domain threshold is between 2 and 16. The peak occurs at rare domain threshold 4 where 37.8% of the proteins have mixed architecture. Note that the concept-based scoring method proposed in this paper does not require or depend on the specification of a rare domain threshold. The purpose of the analysis is this figure is to provide *prima facie* evidence for the feasibility of the concept-based scoring method which relies on a strong presence by mixed architecture proteins for a successful outcome.

Figure 4 shows the breakdown of proteins by domain architecture. As the rare domain threshold increases, the proportion of *only-rare proteins*, i.e. proteins comprising only rare domains, increases, while the proportion of only-promiscuous proteins, i.e. proteins comprising only promiscuous domains, decreases. Both only-rare and only-promiscuous proteins may be single- or multi-domain proteins. *Mixed architecture proteins* comprise both rare and promiscuous domains and are thus necessarily multi-domain proteins. The proportion of mixed architecture proteins is influenced by the proportions of rare and promiscuous domains. When the rare domain threshold is between 2 and 16, mixed architecture proteins make up the largest proportion (at least a third) of the protein population in the Riley dataset. The peak occurs at rare domain threshold 4 where 37.8% of the proteins have mixed architecture.

The concept-based scoring method proposed in this paper does not require or depend on the specification of a rare domain threshold. However, since mixed architecture proteins are the workhorse proteins of the proposed method (Results section), it is reassuring to know that mixed architecture proteins are not rare phenomena in the Riley dataset and that there exists a range of rare domain threshold values where at least a third of the proteins have mixed domain architecture. However for the concept-based scoring method to be feasible at least in principle, the mixed architecture proteins also need to cover gold domains.

**Figure 5:**
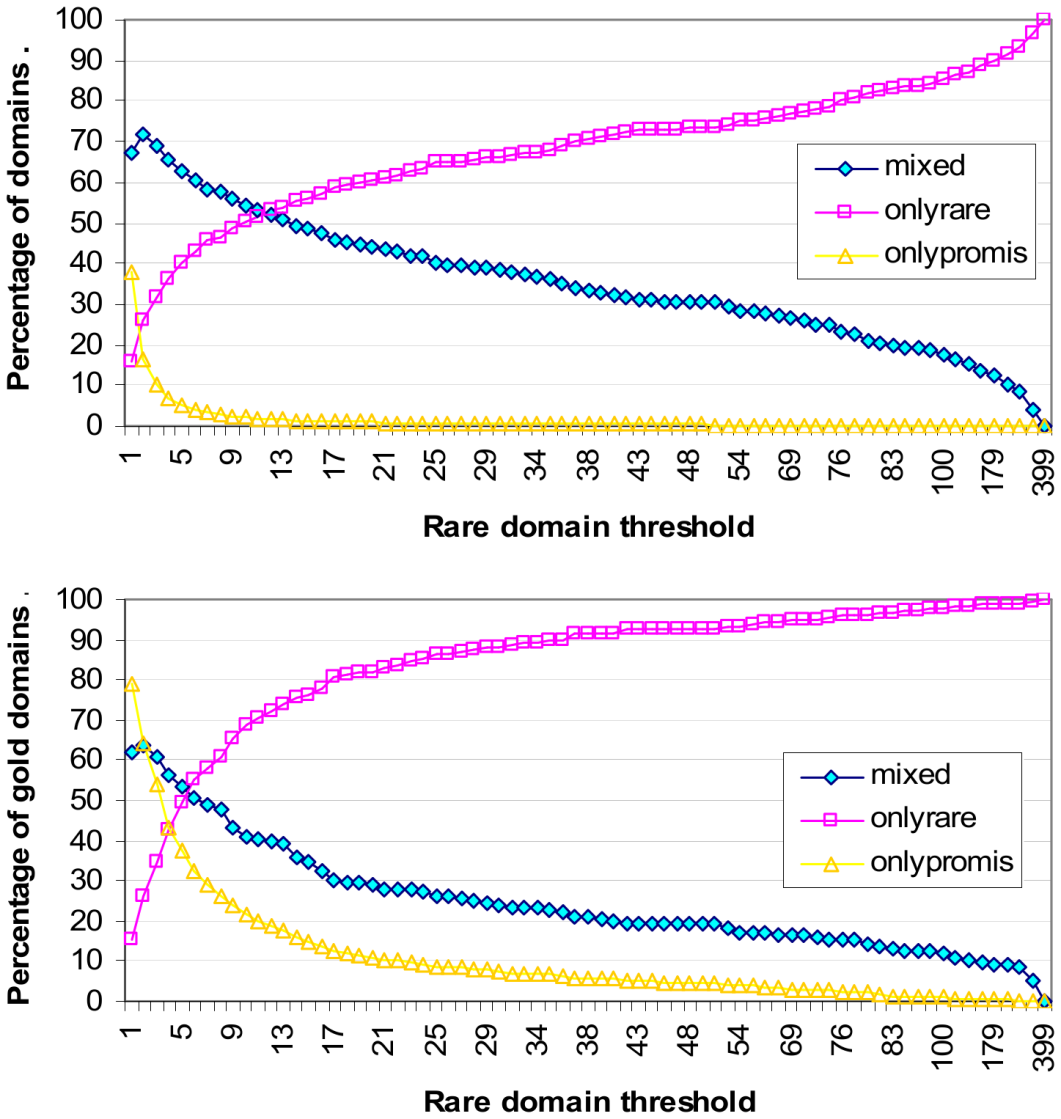
Domain coverage by protein architecture type. Coverage of *all* domains by protein architecture type (top). “onlyrare” refer to proteins comprising one or more rare domains. “onlypromis” refer to proteins comprising one or more promiscuous domains. “mixed” refer to proteins comprising rare and promiscuous domains. Coverage of the 642 *gold* domains by protein architecture types (bottom). A gold domain is a domain that participates in at least one gold standard domain-domain interaction (GDDI). For rare domain threshold values between 2 and 4 inclusive, even though only-rare proteins involve a larger fraction of all domains, they cover a smaller fraction of gold domains than only-promiscuous proteins. Thus gold domains are more likely to be promiscuous domains. Mixed architecture proteins provide the largest coverage of gold domains when the rare domain threshold is between 3 and 5 inclusive. This range lies within the range where mixed architecture proteins make up the largest proportion of the protein population (Figure 4). So there exists a sweet spot where mixed architecture proteins are the most popular protein type and provide the largest coverage of gold domains.

Figure 5 illustrates how domains are covered by proteins of different architectural types. At rare domain threshold 2 and above, only-rare proteins cover a larger portion of all domains than only-promiscuous proteins (Figure 5 top). However, the coverage of gold domains by only-rare proteins exceeds the coverage of gold domains by only-promiscuous proteins only when the rare domain threshold is 5 and larger (Figure 5 bottom). Thus for rare domain threshold values between 2 and 4 inclusive, even though only-rare proteins involve a larger fraction of all domains, they cover a smaller fraction of gold domains than only-promiscuous proteins. This agrees with the notion that gold domains are more likely to be promiscuous.

Up to and including rare domain threshold 11, proteins with mixed architecture provide the largest coverage of all domains (Figure 5 top). However, mixed architecture proteins provide the largest coverage of gold domains only when the rare domain threshold is between 3 and 5 inclusive (Figure 5 bottom). This range lies within the range where mixed architecture proteins make up the largest proportion of the protein population (Figure 4). So there exists a sweet spot where mixed architecture proteins form the most popular protein type and have the largest coverage of gold domains.

## Method

### Formal Concept Analysis

Formal Concept Analysis (FCA) [9] is a technique to organize a (finite) set of objects *G* by their common attributes and dually a (finite) set of attributes *M* by their common objects into a (finite) set of partially ordered pairs of sets called (formal) *concepts*. Implicit is a binary relation *I* ⊆ *G* x *M* which associates objects with attributes. (*g*, *m*) ∈ *I* or equivalently *g I m* denotes object *g* has attribute *m*. The triplet (*G*, *M*, *I*) forms the (formal) *context* within which a FCA is carried out. For small finite examples, a context can be specified completely with a cross-table. The resulting set of concepts, denoted *B*(*G*, *M*, *I*), forms a *concept lattice*.

**Figure 6:**
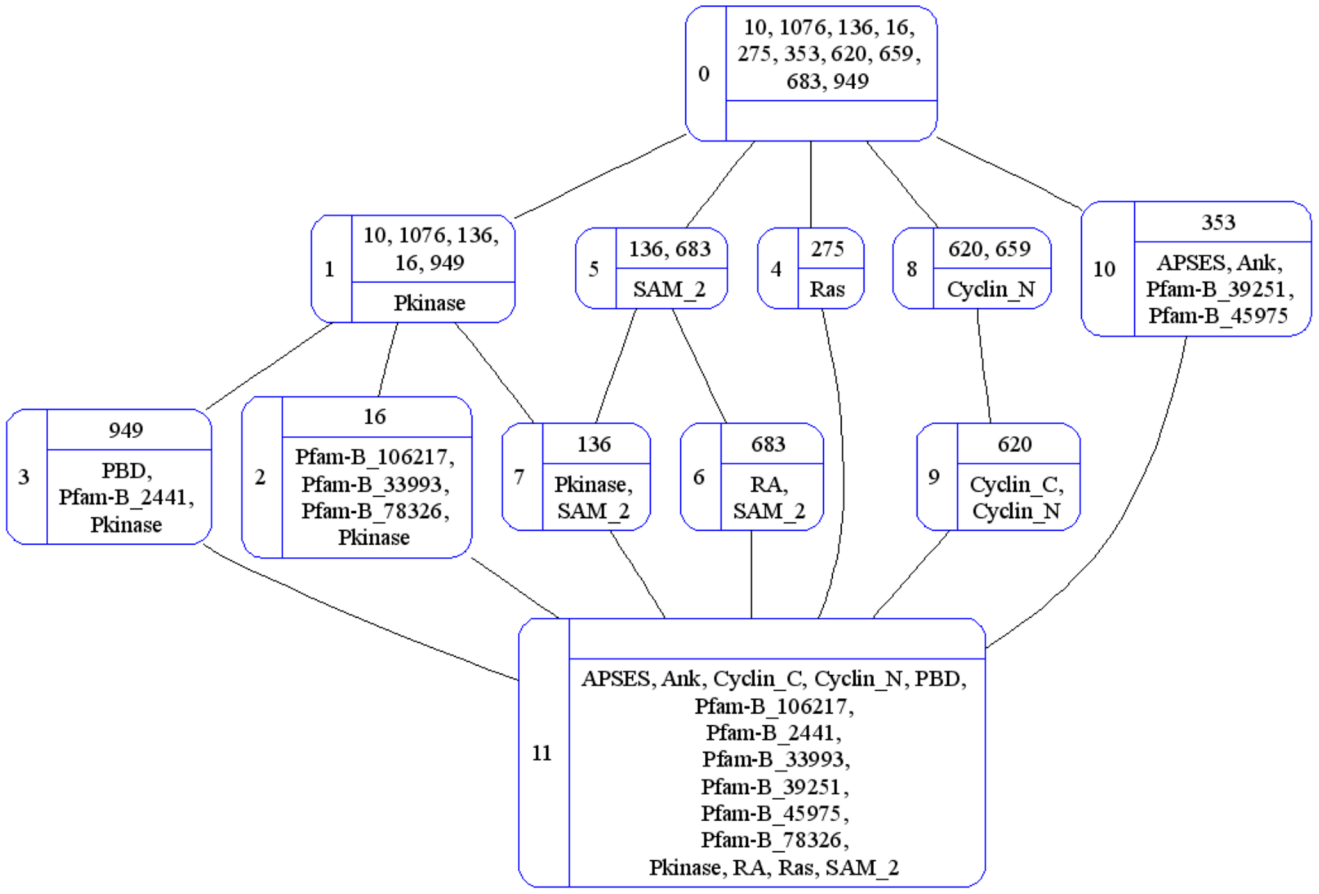
The OA (fully-labeled) concept lattice for the *S. pombe* context in Table 2.

For the application in this paper, the set of objects is the set of proteins, i.e. *G = P*, the set of attributes is the set of domains, i.e. *M* = *D*, and *g I m* denotes protein *g* has domain *m*, i.e. *m* ∈ D(*g*) and dually *g* ∈ P(*m*). Table 2 is the cross-table for the relation between proteins and domains associated with the organism *S. pombe* in the Riley dataset. The (fully-labeled) concept lattice depicting this context is given in Figure 6.

**Table 2:** A cross-table representing the relation between proteins and domains associated with *S. pombe* in the Riley dataset.

	Objects = Proteins (uid)										Domain Freq.
	10	1076	136	16	275	353	620	659	683	949	
Attributes = Domains											
APSES						x					1
Ank						x					1
Cyclin_C							x				1
Cyclin_N							x	x			2
PBD										x	1
Pfam-B_106217				x							1
Pfam-B_2441										x	1
Pfam-B_33993				x							1
Pfam-B_39251						x					1
Pfam-B_45975						x					1
Pfam-B_78326				x							1
Pkinase	x	x	x	x						x	5
RA									x		1
Ras					x						1
SAM_2			x						x		2
Domains per protein	1	1	2	4	1	4	2	1	2	3	

E.g.: the domain set for protein 353, D(353) = {APSES, Ank, Pfam-B_39251, Pfam-B_45975}; and the protein set for domain Pkinase, P(Pkinase) = {10, 1076, 136, 16, 949}.

#### FCA in more detail

This section describes FCA for those unfamiliar with the theory and in enough detail to support the discussion in this paper. The more mathematically inclined are referred to [9] for a rigorous and complete exposition of FCA.

A concept *c* ∈ *B*(*G*, *M*, *I*) is an ordered pair of sets (*O*, *A*) such that *O* ⊆ *G*, *A* ⊆ *M* and the set of all attributes common to all objects in *O* under relation *I* is *A* and the set of all objects with attributes in *A* under relation *I* is *O*. More formally, the last two conditions are *A* = *O′* = {*m* ∈ *M* | (µ*g* ∈ *O*) *g I m*} and *O* = *A′* = {*g* ∈ *G* | (µ*m* ∈ *A*) *g I m*} respectively. If this seems a bit chicken-and-egg, these last two conditions can be satisfied by working from the power-set of objects or alternatively from the power-set of attributes; but there exist more efficient FCA algorithms such as Lindig’s C implementation called *Colibri-concepts* [12] which is freely available on-line and runs on Linux. For the complete protein-domain relation in the Riley dataset, Colibri-concepts produced a lattice with 8,894 concepts in less than 5 minutes on a Linux machine allocated with a maximum of 2 Gbs of memory.

The set of objects *O* is called the *extent* of concept *c*, and the set of attributes *A* is known as the *intent* of concept *c*. A concept’s extent is denoted O(*c*) and its intent as A(*c*). The prime symbol *′* denotes the mapping from an extent to its intent and vice versa, i.e. let a concept *c* = (*O*, *A*), then *O′* = *A*, *A′* = *O*, *O* = *O″*, *A* = *O″′*, and so on. This pair of maps between the set of extents and the set of intents forms a Galois connection between the two partially ordered sets. The statements *O* = *O″* and *A* = *O″′* are true due to the maximal condition for extents and intents. This implies that if a set of objects (attributes) forms the extent (intent) of a concept, then the set of objects (attributes) uniquely identifies the concept, and conversely a concept unambiguously identifies its extent and its intent.

The set of concepts is ordered by set inclusion 〈*B*(*G*, *M*, *I*); ≤〉. For two distinct concepts in *B* (*G*, *M*, *I*), (*O*_1_, *A*_1_) ≤ (*O*_2_, *A*_2_) implies *O*_1_ ⊂ *O*_2_ and dually *A*_1_ ⊃ *A*_2_. The join (least upper bound) and meet (greatest lower bound) are defined for every pair of non-comparable concepts in 〈*B*(*G*, *M*, *I*); ≤〉. 〈*B*(*G*, *M*, *I*); ≤〉 forms a concept lattice. Intuitively, a concept lattice is a two-in-one lattice with a right-side up lattice for the set of extents and an upside down lattice for the set of intents. More formally, a concept lattice is a complete lattice with a top element (*G*, ∅) and a bottom element (∅, *M*). A complete lattice defined on a subset of a power-set is closed under arbitrary joins (in the form of unions) and meets (in the form of intersections) [13]. Within a concept lattice, the join (supremum) of two arbitrary concepts *c*_1_ ∨ c_2_ =  ((O(*c*_1_) ∪ O(c_2_))*″*, A(*c*_1_) ∩ A(c_2_) ), and the meet (infimum) of two arbitrary concepts c_1_ ∧ c_2_ =  ((O(*c*_1_) ∩ O(c_2_), (A(*c*_1_) ∪ A(c_2_))*″* ). *c*_1_ ∨ c_2_ is a concept since A(*c*_1_) ∩ A(c_2_) =  (O(*c*_1_) ∪ O(c_2_))′ and (*O″*, *O′*) is always a concept. Similarly, *c*_1_ ∧ c_2_ is a concept since O(*c*_1_) ∩ O(c_2_) = (A(*c*_1_) ∪ A(c_2_))′ and (*A′*, *A″*) is always a concept. The intersection of any number of extents (intents) always results in an extent (intent). The same is not generally true for unions of extents (intents) [9]. Rather, (O(*c*_1_) ∪ O(c_2_)) ⊆ (O(*c*_1_) ∪ O(c_2_))*″* and (A(*c*_1_) ∪ A(c_2_)) ⊆ (A(*c*_1_) ∪ A(c_2_))*″* hold.

**Figure 7:**
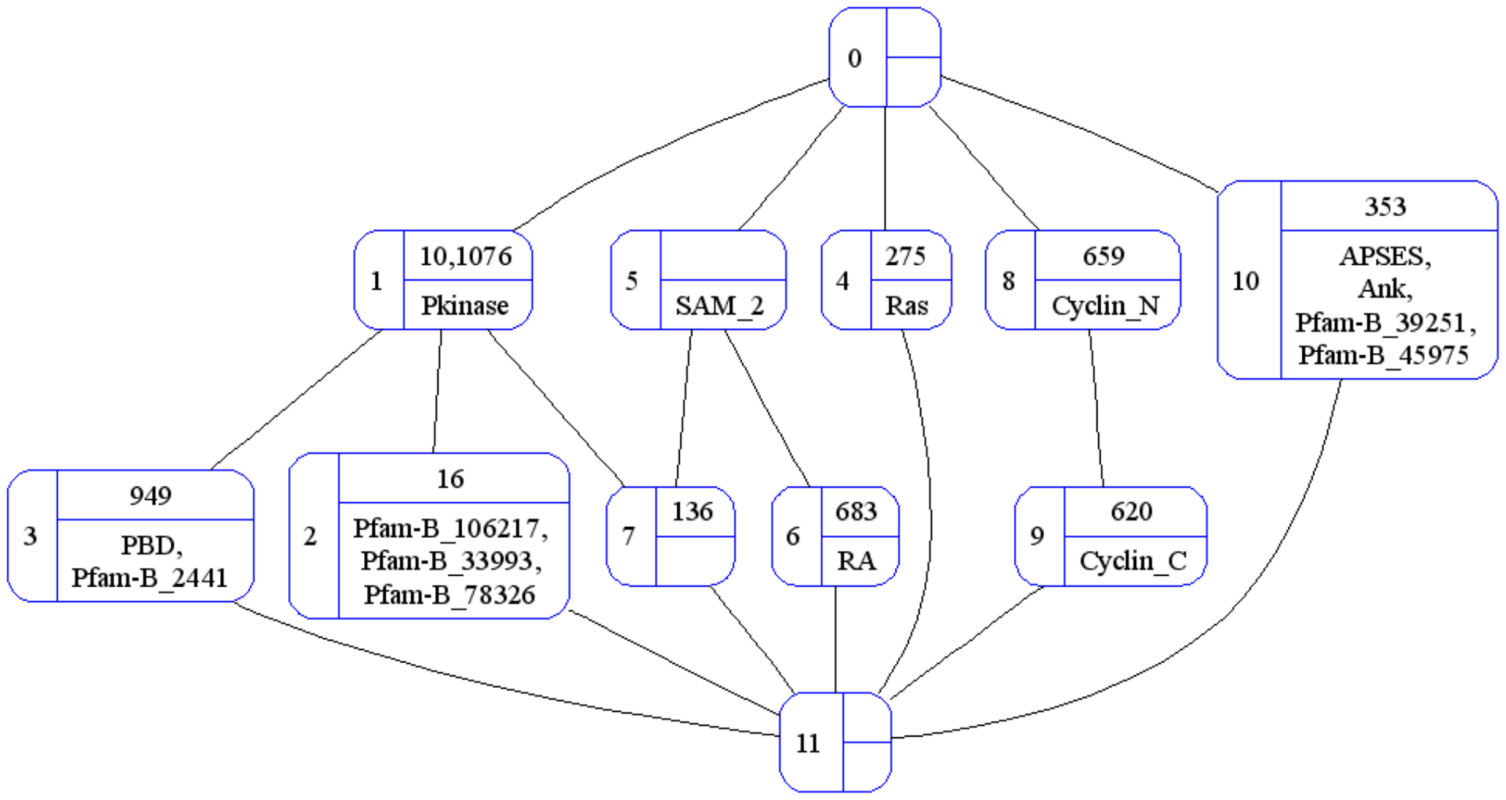
The oa (reduced) concept lattice for the context in Table 2. The **OA** concept lattice was presented in Figure 6. The **oA** and **Oa** concept lattices are given in Figures S2 and S3 in File S1, respectively.

The down-set of *c* represented as ↓{*c*} = {*x* ∈ 〈B(*G*, *M*, *I*); ≤〉 | *x* ≤ *c*}. The up-set of *c* represented as ↑{*c*} = {*x* ∈ 〈*B*(*G*, *M*, *I*); ≤〉 | *c* ≤ *x*}. The extent of a concept *c* is the union of the extent of each concept ∈ ↓{*c*}. The intent of a concept *c* is the union of the intent of each concept ∈ ↑{*c*}. This relationship between concepts makes it possible to reduce the labeling of concepts to objects and attributes specific to a concept (Figure 7). Changing the labels does not change the concepts. A concept lattice with reduced labeling is a *reduced concept lattice*.

There are then four possible ways to label a concept lattice: (i) with complete object labels and complete attribute labels (**OA**); (ii) with reduced object labels and reduced attribute labels (**oa**); (iii) with complete object labels and reduced attribute labels (**Oa**); and (iv) with reduced object labels and complete attribute labels (**oA**). The **oa** and **oA** combinations produce *object-reduced concept lattices*. The **oa** and **Oa** combinations produce *attribute-reduced concept lattices*. In an object-reduced concept lattice, object labels appear exactly once and the set of object labels (proteins) is finite. Similarly in an attribute-reduced concept lattice, attribute labels appear exactly once and the set of attribute labels (domains) is finite. Each combination is explored in this paper for the proteins and their domains in the Riley dataset. It is possible for two different combinations to produce two different outcomes because the labels of objects and attributes are used instead of the objects and attributes themselves. O^L^(*c*) refers to the set of object-labels for O(*c*), and similarly A^L^(*c*) refers to the set of attribute-labels for A(*c*).

#### Organization of domains and proteins in a concept lattice

Promiscuous domains gravitate towards the top of an attribute-reduced concept lattice. This is expected since for a domain to be promiscuous, it must occur in many proteins. In FCA language, involving more proteins (objects) means a larger extent, and as one goes up in a concept lattice extents increase in size, culminating in the top element whose extent is the entire object set. For *S. pombe*, the domains with frequency N(*d*) >1 are Pkinase (5), SAM_2 (2) and Cyclin_N(2) (Table 2) and they reside in concepts one step away from the top element but two steps away from the bottom element (Figure 7). In contrast, the rare (non-promiscuous) domains gravitate towards the bottom of an attribute-reduced concept lattice since by their rarity, rare domains command smaller extents.

The position of a protein in an object-reduced concept lattice depends on the promiscuity of its domain(s). Three of the four single-domain proteins for *S. pombe* have promiscuous domains (N(*d*) >1), and these single-domain proteins (10, 1076 and 659) reside in concepts one step away from the top element in Figure 7. A multi-domain protein with a combination of promiscuous and rare domains will appear in an object-reduced concept lattice with its rare domains. E.g. protein 620 appears with domain Cyclin_C and not with Cyclin_N (Figure 7). Also in Figure 7, protein 16 appears with its Pfam-B domains (which tend to be rare) in concept 2 and not with the more promiscuous Pkinase in concept 1. In general therefore, proteins with rare domains will gravitate towards the bottom of an object-reduced concept lattice, and proteins with only promiscuous domains will gravitate towards the top of an object-reduced concept lattice. Further, because attributes accumulate downwards in a concept lattice, concepts containing multi-domain proteins will tend to be sub-concepts of concepts containing single-domain proteins. These points are illustrated in Figure S4, Figure S5, and Table S1 in File S1.

In a reduced concept lattice, there will be concepts with an empty object-label set or an empty attribute-label set. At the very least the object-label sets and attribute-label sets of the top and bottom elements will be, by definition, empty. The more numerous rare domains will “consume” proteins and leave fewer proteins available to fill the object-label sets of concepts with promiscuous domains. Thus an empty object-label set O^L^(*c*) = ∅ is more likely towards the top of an object-reduced concept lattice. Concepts with multi-domain protein(s) in their extents will tend to have empty attribute-label sets in an attribute-reduced concept lattice unless the multi-domain protein(s) introduces “new” domains. E.g. concept 7 in Figure 7 has an empty attribute-label set since the domain set it “inherits” is {Pkinase, SAM_2} which is exactly the domains of protein 136. In contrast, the attribute-label set of concept 2 is not empty but is filled with domains specific to protein 16. Hence, empty attribute-label set A^L^(*c*) = ∅ is more likely towards the bottom of an attribute-reduced concept lattice. The presence of empty object-label sets and empty attribute-label sets influences the number of concept-pairs available to evaluate domain-pairs, and the diversity of CB and PG values produced by the concept-based scoring method.

### Domain-pair Scoring and Ranking

One of the earliest methods for detecting over-represented ‘correlated sequence-signatures’ e.g. domain-pairs, in a database of protein-protein interactions used the log-odds of the ratio between observed and expected frequencies to score pairs of sequence-signatures [14]. Larger scores indicate a frequency of occurrence in the database which is higher than expected by random chance. This method was called the Association method in [1] and subsequently adopted in other papers, e.g. [6].

Specifically, the score of a domain-pair (*a*, *b*) with the Association method is AM(*a*, *b*) =  log_2_ [M(*a*, *b*)/(N(*a*) × N(*b*))]. M(*a*, *b*) = | {(*x*, *y*)^1^ | *a* ∈ D(*x*) and *b* ∈ D(*y*)} | is the number of interacting protein-pairs in the database such that *a* is a domain of protein *x* and *b* is a domain of protein *y*. N(*d*) is the number of proteins in the database that has domain *d* in its domain architecture. The scores are negative in value with a maximum of log_2_(1) = 0 which is the score for domain-pairs that occur only between interacting protein-pairs, and an undefined minimum of log_2_(0) which is the score for domain-pairs that occur only between non-interacting protein-pairs. Domain-pairs with larger scores are ranked more highly.

The Riley dataset comprises proteins from multiple organisms, and both interacting and non-interacting proteins are restricted to those from the same organism. To handle this situation, AM(*a*, *b*) = log_2_ [M(*a*, *b*)/(M(*a*, *b*)+Z(*a*, *b*))]. M(*a*, *b*) is defined previously. Z(*a*, *b*) = | {(*x*, *y*)^0^ | *a* ∈ D(*x*) and *b* ∈ D(*y*)} | is the number of non-interacting protein-pairs in the database such that *a* is a domain of protein *x* and *b* is a domain of protein *y*.

### Concept-based Scoring and Ranking <CB, PG>

**Figure 8:**
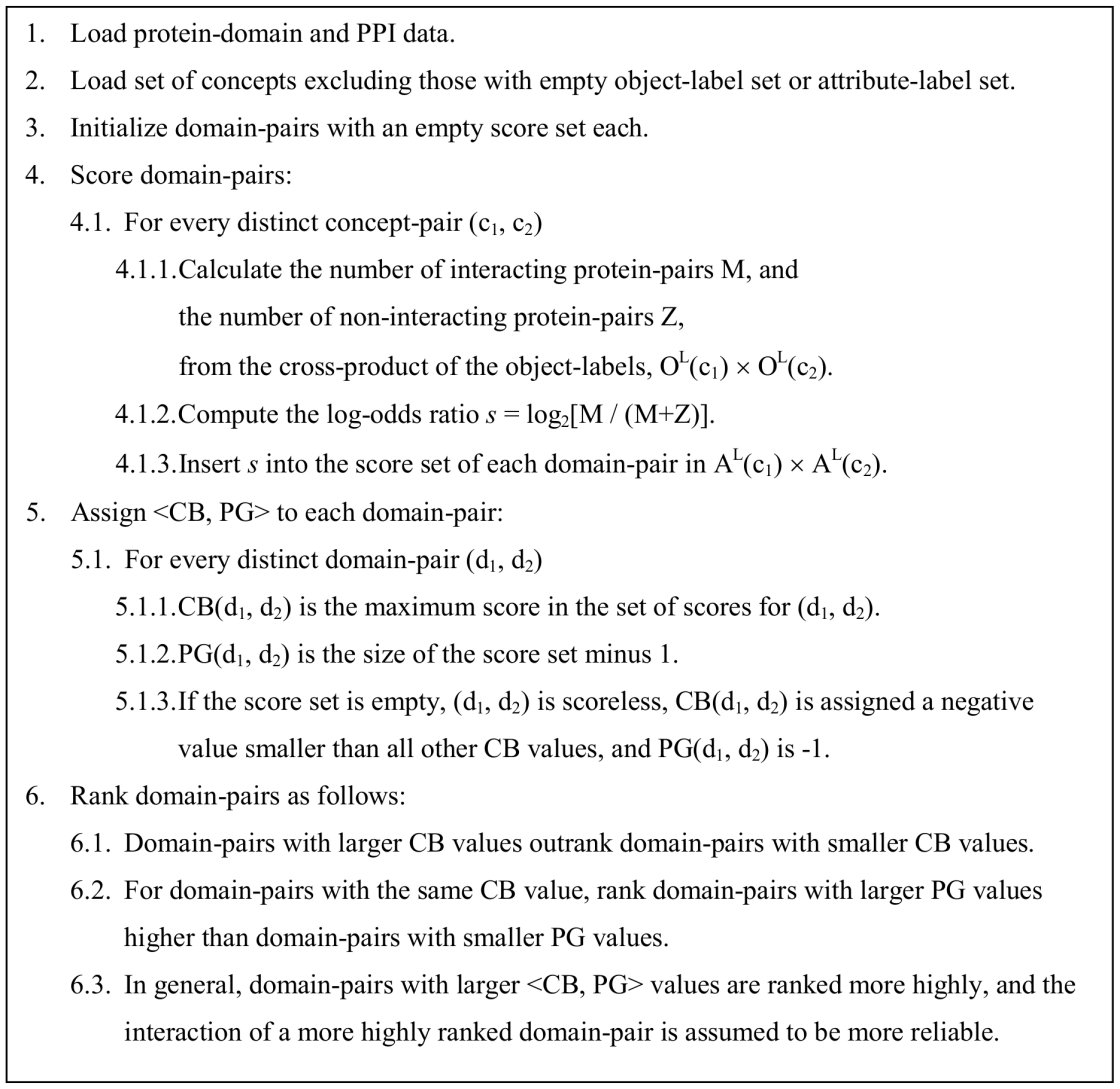
Key steps in the proposed concept-based scoring and ranking method for domain-pairs. See text for further explanation and Figures S6 and S9 in File S1 for a walk-through on how to compute the <CB, PG> value for a domain-pair.

The *concept-based scoring* scheme proposed in this paper (Figure 8) also uses the log-odds ratio AM(*a*, *b*) described before, but the scoring is done using pairs of concepts. The concepts used exclude the top and the bottom concepts, and any other concepts with an empty object-label set or an empty attribute-label set. Protein-pairs generated by the object-label sets of a concept-pair, i.e. O^L^(*c*_1_) × O^L^(*c*_2_), are used to compute the log-odds ratio which is then used to evaluate the domain-pairs generated by the attribute-label sets of said concept-pair, i.e. A^L^(*c*_1_) × A^L^(*c*_2_). Essentially, each concept-pair (*c*_i_, *c*_j_) provides a different context (in the form of protein-pairs) to evaluate a domain-pair. The set of concept pairs {(*c*_i_, *c*_j_)} used to evaluate a domain-pair (*a*, *b*) is determined by the attribute-label sets, and comprises all distinct concept-pairs where *a* ∈ A^L^(*c*_i_) and *b* ∈ A^L^(*c*_j_). Concept-based scoring and ranking is demonstrated in Figures S6, S7, S8 and S9, and Table S2 in File S1.

With concept lattices that are not attribute-reduced (**oA** and **OA**), a domain-pair can have more than one score. CB(*a*, *b*) is the largest score found for domain-pair (*a*, *b*), while PG(*a*, *b*) is the number of unique scores for (*a*, *b*) which are strictly smaller than CB(*a*, *b*). Domain-pairs with larger CB scores are ranked more highly. The PG scores help to break ties between domain-pairs with identical CB scores. Given identical CB scores, domain-pairs with larger PG scores are ranked more highly.

A domain-pair may be left scoreless if it was not evaluated at all (this is possible with the **oa** concept lattice when many concepts are excluded because of empty object-label or empty attribute label sets), or if its evaluation results in an undefined score, i.e. log_2_(0). Scoreless domain-pairs are given a negative PG value, and are placed in random order below domain-pairs with CB scores. The **Oa** scores are identical to those obtained with the Associative method since every domain appears exactly once in an **Oa** concept lattice and all proteins containing a domain appears in the object-label set of a concept whose attribute-label set has the domain.

**Figure 9:**
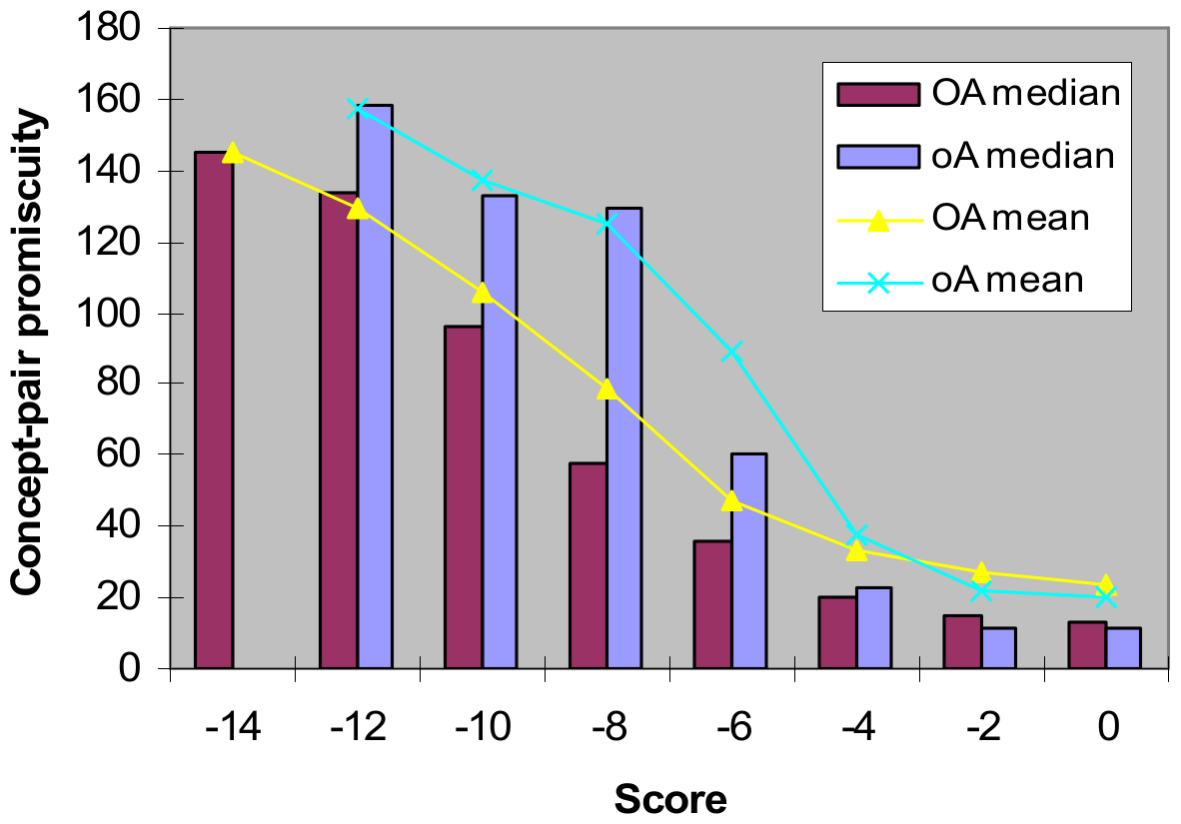
Concept-pair promiscuity decreases as score increases. Spearman’s rank correlation rho for **OA** is −0.4934008, and for **oA** is −0.3893343. Promiscuity of a domain-pair (a, b) is [N(*a*)+N(*b*)]/2 where N(*d*) is the number of times domain *d* occurs in a set of proteins. For a concept-pair (*c_i_*, *c_j_*), promiscuity is the average promiscuity of all domain-pairs in A^L^(*c_i_*) × A^L^(*c_j_*), and the score is the log-odds ratio of interacting protein-pairs to non-interacting protein-pairs in O^L^(*c_i_*) × O^L^(*c_j_*). For **OA** concept-pairs that produce a score in [−12, −10), the median promiscuity is 133.5 and the mean promiscuity is 129.2; when the score is 0.0, the median promiscuity falls to 13.25 and the mean promiscuity is 23.47. There were no **oA** scores smaller than −12.0. In non-attribute-reduced concept lattices (**OA** and **oA**), a domain-pair can be generated by more than one concept-pair (*c_i_*, *c_j_*) through the cross-product of their attribute-label sets, i.e. A^L^(*c_i_*) × A^L^(*c_j_*). This paves the way for a more promiscuous domain-pair to have the same score as a less promiscuous domain-pair. The *piggy-backing mechanism* takes effect when a domain-pair improves its score because either one or both of its domain partners happen to occupy the same attribute-label set as one or more rarer domains.

The number of times a domain-pair is evaluated depends on the promiscuity of the domain-pair and the type of concept lattice used. A domain-pair can be evaluated at most once in an attribute-reduced concept lattice. When the concept lattice type permits (i.e. attribute-labels are not reduced), domains that are more promiscuous have more opportunity to appear in different attribute-label sets. The question then becomes is it better for a promiscuous domain to appear with other promiscuous domains or with rare domains. The results suggest that a promiscuous domain-pair is more likely to improve its score if either one or both of its partner domains appear with rare domains. This is because rare domains tend to produce fewer non-interacting protein-pairs. The *piggy-backing mechanism* takes effect when a domain-pair improves its score because one or both of its domain partners happen to occupy the same attribute-label set as one or more rarer domains. Evidence of piggy-backing in non-attribute-reduced concept lattices is supported by the presence of positive PG values, and a negative correlation between score and average promiscuity of domain-pairs. This negative correlation can be observed when evaluating individual domain-pairs (e.g. in Figures S6 and S9 in File S1), and also in general over all concept-pairs (Figure 9). The facility for promiscuous domains to appear with rare domains in the same attribute-label set is provided by mixed architecture proteins.

Thus, several conditions favourable to the concept-based scoring method arise:

- The domain-pairs that need to be ranked highly are promiscuous. The GDDIs are promiscuous.
- It must be possible to evaluate a domain-pair with different protein-pairs. This is possible only with concept lattices that are *not* attribute-reduced, i.e. **OA** and **oA**. Strictly speaking then, neither **oa** nor **Oa** can produce concept-based scores since their PG values are always ≤ 0. The **Oa** scores are identical to those of the Association method.
- To improve the score of promiscuous domain-pairs through piggy-backing, it must be possible for promiscuous domains to reside with rare domains in one or more attribute-label sets. This is made possible by multi-domain proteins, in particular those which have a mix of promiscuous and rare domains in their architecture, i.e. mixed architecture proteins. Within a sensible range of rare domain thresholds, a substantial proportion of proteins in the Riley dataset are proteins with mixed architecture (Figure 4) and these proteins cover a substantial proportion of the gold domains (Figure 5).

## Results

The concept-based scoring method is applied to the Riley dataset using all four types of concept lattices. The algorithm in Figure 8 was implemented in C++ and compiled with g++ -O3. No special effort was made to optimize the code. With the given dataset, the program ran to completion in under 15 minutes with 2 GB of memory on a 64-bit Linux machine. The domain-pair rankings are evaluated on three fronts: (i) Correlation between rank and promiscuity of GDDIs, (ii) GDDI recovery and (iii) the Nye test [15]. The hypothesis is that when conditions are favourable to piggy-backing, i.e. the concept lattice is not attribute-reduced and a good proportion of proteins are of mixed architecture, highly ranked DDIs are expected to be more promiscuous. Since GDDIs are more promiscuous than non-GDDIs, highly ranked domain-pairs will be enriched with GDDIs. This in turn will aid GDDI recovery and increase the pass rate on the Nye test.

### Four Evaluation Scenarios

The concept-based scoring method is evaluated under the following four circumstances, all of which are related to input data characteristics. In all scenarios, R’s Wilcox.test confirms that the set of GDDIs is still significantly more promiscuous than the set of non-GDDIs. More promiscuous GDDIs are more likely to survive the changes. GDDIs make up 0.44% of DDIs in **A**, 0.60% in **B**, 0.21% in **C** and 0.11% in **D**. Median promiscuity of GDDIs to DDIs is 7.0∶5.0 in **A**, 8.5∶5.5 in **B**, 9.5∶5.5 in **C** and 15.0∶4.0 in **D**. With a smaller GDDI to DDI percentage and a much larger GDDI to DDI promiscuity ratio, scenario **D** is the most difficult of all. The resultant number of PPIs, DDIs, GDDIs and GPPIs for each scenario is summarized in Table S3 in File S1.

- Under the default or original circumstance, the complete Riley dataset is used without modification. Since all PPIs in the given input data are used, the probability of including a PPI, Pe is 1.0.
- PPI data obtained via high-throughput methods are error-prone. To account for the inaccuracies in PPI data, the robustness of computational methods when dealing with PPIs is commonly tested by using Pe <1.0. Ref. [6] for example, reported the results for their method at Pe = 0.5. The concept-based scoring method is also evaluated at Pe = 0.5, that is each PPI from the set of PPIs in the Riley dataset is included with 50% probability.
- The log-odds ratio depends on the set of protein-pairs used and also on the PPI network. To test the robustness of the results against changes in PPIs, the nodes of each organism’s PPI network were shuffled amongst themselves. Node shuffling generates a new set of PPIs, but the number of PPIs per organism and the original PPI network structure (e.g. degree distribution, average path length, clustering and degree-degree assortativity) remains unchanged. The new set of DDIs overlaps but is no longer a subset of the original DDIs.
- Finally, to test the influence of domain architecture on the results, the domains of proteins are shuffled. For this scenario domain repetition within a protein is allowed and a protein becomes a multi-set of domains. The shuffle changes the frequency of domains in a small but still statistically significant way. The new set of DDIs overlaps but is no longer a subset of the original DDIs. Domain shuffling was accomplished with the following steps:
  1. Place every instance of a domain in the input data into a sequence, sorted by frequency of occurrence. Domain instances with identical frequency are shuffled amongst themselves.
  2. Sort the proteins by their size, i.e. number of domains they contain.
  3. Starting from the largest to the smallest protein, assign domains to proteins starting from the least to the most frequently occurring domains. The reason for this is to reduce domain repetition within a protein.

**Figure 10:**
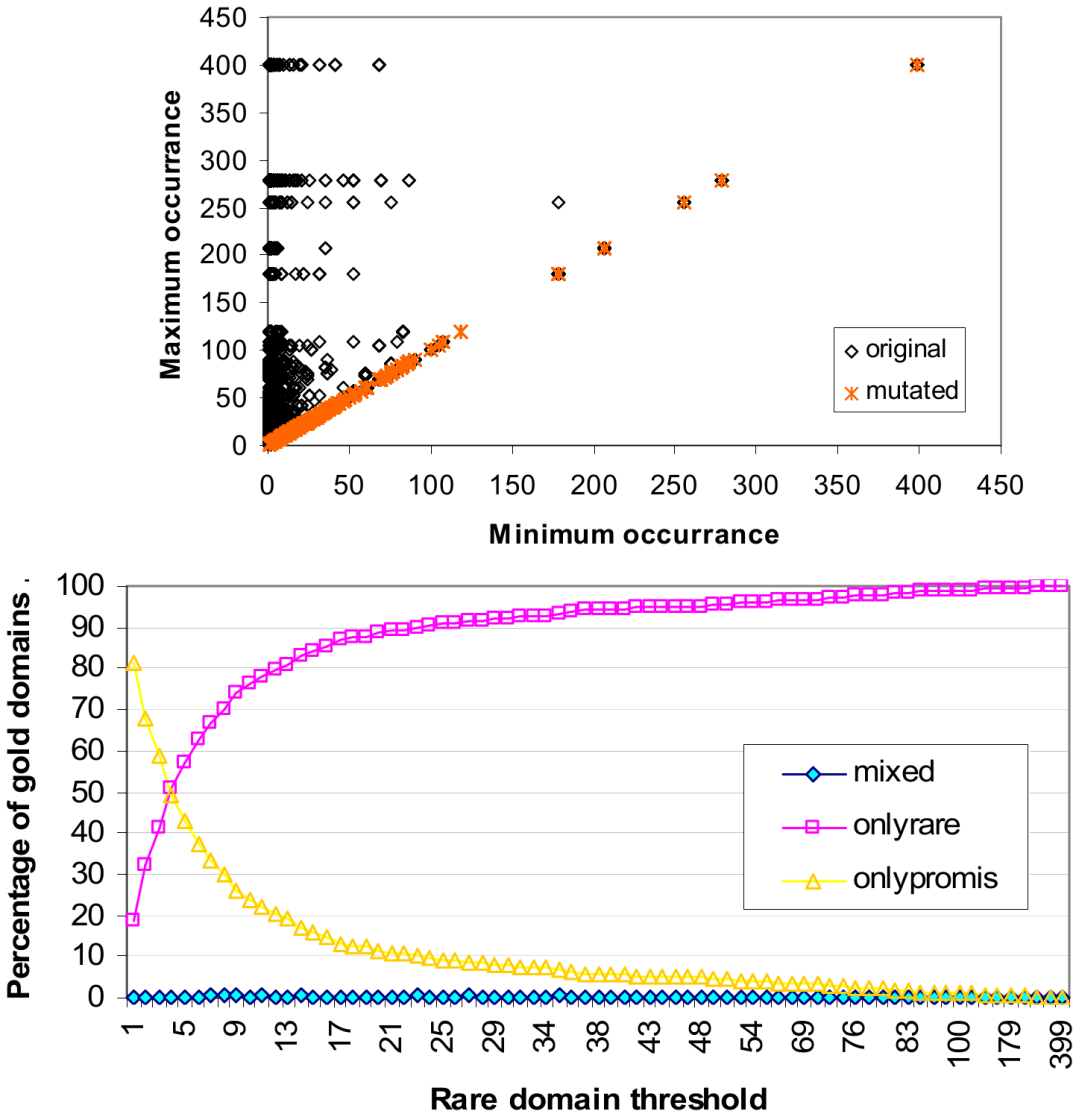
Impact of domain shuffling on domain architecture (top). Each point plots the minimum and maximum domain frequency in a protein. For example, the original domain set for protein 949, D(949) = {PBD, Pfam-B_2441, Pkinase} (Table 2). The minimum and maximum occurrence values for D(949) are 1 and 5 respectively. A point (x, y) in the plot denotes the minimum and maximum domain occurrence in a protein. Prior to domain shuffling, there are proteins with both rare and promiscuous domains, as shown by the black markings in the upper left of the plot. After domain shuffling, proteins have domains which are either rare only or promiscuous only, as shown by the orange markings on the y = x line. Domain shuffling changes the original heterogeneous domain architecture to a homogeneous one in terms of domain occurrence. **Gold domain coverage by protein type after domain shuffling (bottom).** At all rare domain threshold values, at least 99.5% of the 642 gold domains are covered by either proteins comprising only rare domains or proteins comprising only promiscuous domains. When the rare domain threshold is 4, the coverage comes very close to a 50∶50 split. Compare with Figure 5 (bottom) for gold domain coverage by protein type before domain shuffling.

The impact of shuffling on domain architecture is shown in Figure 10 (top) which plots the minimum and maximum domain occurrence for each protein. Points from the original domain architecture (prior to domain shuffling) concentrate on the left half of the plot, while points from the shuffled (mutated) domain architecture occupy the y = x line. This reveals that mixed domain architecture in proteins is destroyed by the shuffling. The homogeneity in the mutated proteins is because protein sizes are shorter than the length of a subsequence of domains with identical occurrence. Gold domain coverage by protein type after shuffling is given in Figure 10 (bottom). At all rare domain threshold values, at least 99.5% of the 642 gold domains are covered by either proteins comprising only rare domains or proteins comprising only promiscuous domains. When the rare domain threshold is 4, the coverage comes very close to a 50∶50 split. Compare with Figure 5 (bottom) for gold domain coverage by protein type before domain shuffling. Domain shuffling changes the context between proteins and domains. As such, a new concept lattice is computed.

### Correlation between GDDI Rank and Promiscuity

**Figure 11:**
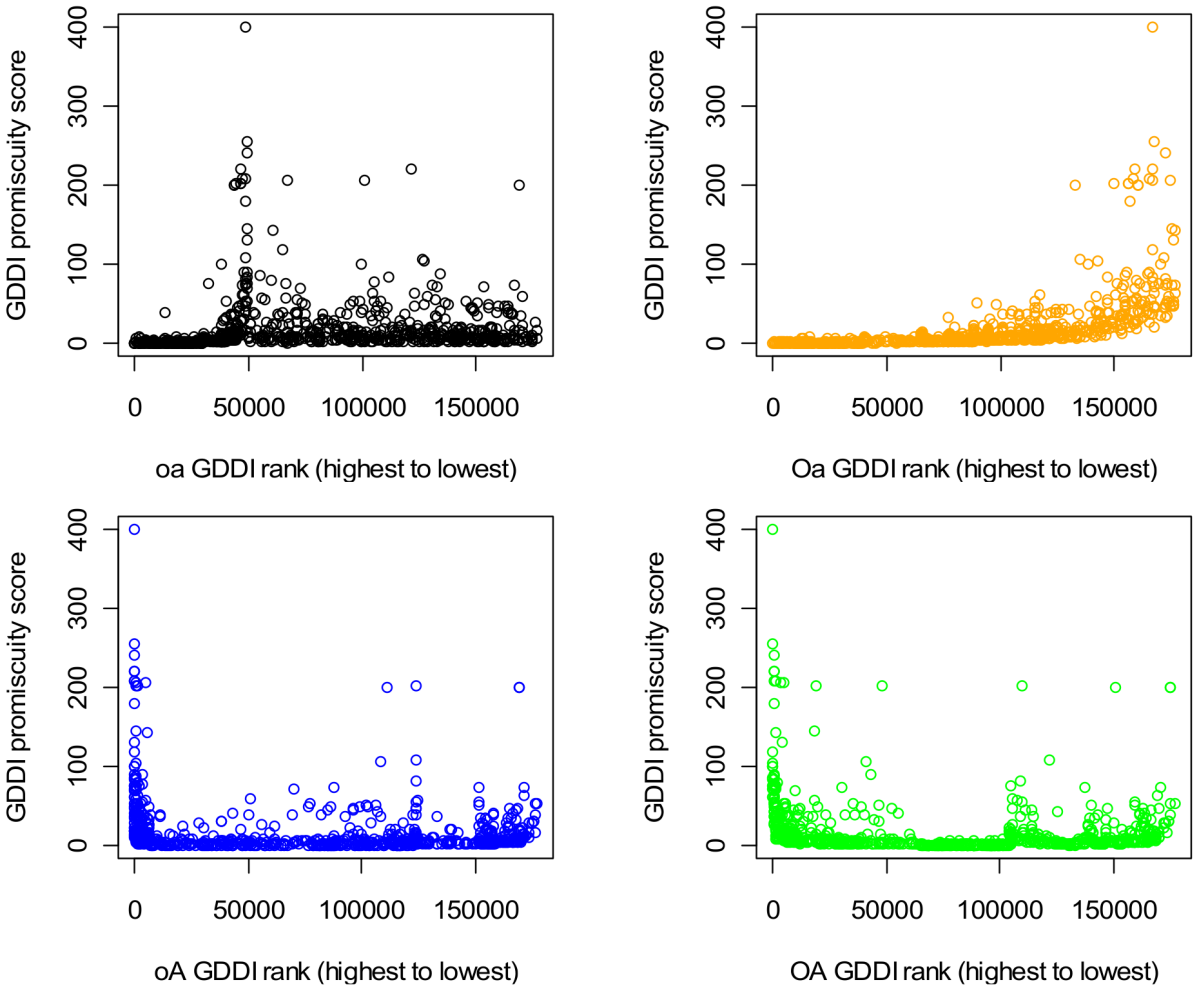
Scatter-plot of GDDI rank vs. promiscuity, scenario A Pe = 1.0. All the 177,233 putative DDIs were ranked as described in the text, and the ranks of GDDIs were extracted to create the plots. Promiscuity of a domain pair (*a*, *b*) = [N(*a*)+N(*b*)]/2 where N(*d*) is the number of times domain *d* occurs in a protein set. Only the concept lattices which are not attribute-reduced (**OA** and **oA**) exhibit the desired negative relationship, which means they tend to rank promiscuous GDDIs more highly. The relationship is strongly positive when the **Oa** rankings are used. **Oa** results are identical to the Associative method which is known to penalize promiscuous domain-pairs. There is also a tendency for the **oa** concept lattice to rank promiscuous GDDIs less highly, but this positive relationship is not so apparent because scoreless GDDIs are included in the plot (they start at rank 49,378 and onwards to the right of the plot). When the **oa** concept lattice is used, only 350 of the 783 GDDIs have CB scores; the remaining GDDIs are scoreless and are ranked randomly but below the GDDIs with CB scores. The same pattern of relationships is observed with evaluation scenarios **B** and **C** (Figures S11 and S12 in File S1).

Figure 11 plots the promiscuity of GDDIs against their rank obtained with different concept lattice types. Promiscuity of a domain pair (*a*, *b*) = [N(*a*)+N(*b*)]/2 where N(*d*) is the number of times domain *d* occurs in a protein set. The GDDI rankings were extracted from the rankings of all DDIs (ranking domain-pairs is described in step 6 of Figure 8). However, to reduce rank ties a small random element is added to GDDIs with the same <CB, PG> score such that each GDDI has a unique numeric rank value. Ranking starts at 0 and declines as numeric values get larger. GDDIs with larger <CB, PG> scores have higher rank but smaller numerical rank value. For this reason, the expected correlation is a negative one.

Of the four plots in Figure 11, only the **OA** and **oA** ones exhibit a negative correlation between GDDI promiscuity and rank. A negative correlation implies that higher ranking GDDIs tend to be more promiscuous. The relationship is strongly positive (Spearman’s rank correlation rho = 0.88) when the **Oa** rankings are used. **Oa** results are identical to the Associative method which is known to penalize promiscuous domain-pairs.

There is also a tendency for **oa** to rank promiscuous GDDIs less highly (Spearman’s rank correlation rho = 0.49), but this positive relationship is not as strong as **Oa**’s because scoreless GDDIs are included in the plot (they start at numeric rank value 49,378 and above). When the **oa** concept lattice is used, only 350 of the 783 GDDIs have CB scores. The remaining 55.3% of GDDIs which are scoreless are ranked in random order below the GDDIs with CB scores. But R’s Wilcox.test confirms that the domain-pairs (including non-GDDIs) with CB scores are significantly less promiscuous than the scoreless domain-pairs. Hence, the **oa** concept lattice tends to ranks more promiscuous domain-pairs less highly.

The increased presence of empty object-label sets and empty attribute-label sets in a reduced (**oa**) concept lattice is the reason many domain-pairs are scoreless. Concepts with an empty object-label set or an empty attribute-label set are ignored when computing scores for domain-pairs (Figure 8). For the Riley dataset, 127856/177233 = 72.14% of the possible domain-pairs are left scoreless by the **oa** concept lattice. The many scoreless domain-pairs means the **oa** results are unsuitable or too weak for identifying strong interactions in protein complexes. **oa** performs poorly in the Nye test (Results section). Although **oA** has empty object-label sets and **Oa** has empty attribute-label sets, they do not produce any scoreless domain-pairs for the Riley dataset. The number of concepts used by each type of concept lattice for the Riley dataset is given in Table S4 in File S1.

**Figure 12:**
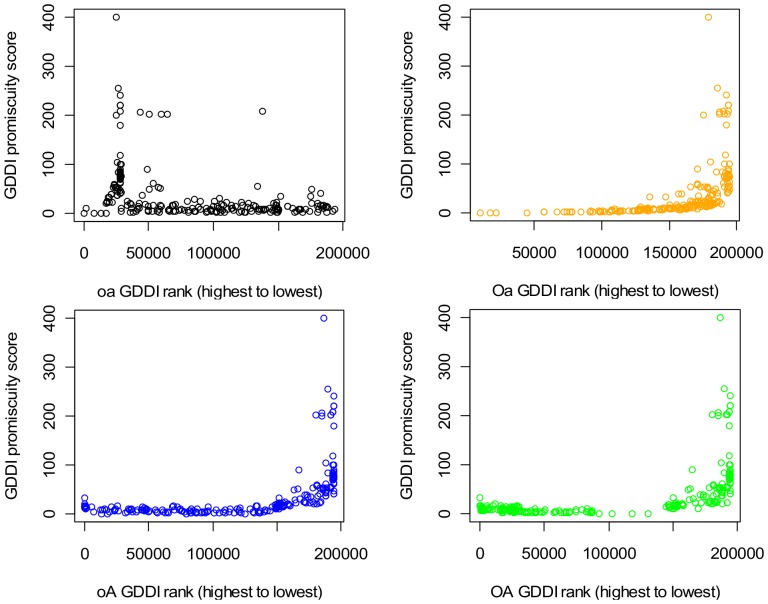
Scatter-plot of GDDI rank vs. promiscuity, scenario D shuffled domains, Pe = 1.0. Mixed architecture proteins play a critical role in the ability of non-attribute-reduced concept lattices to rank promiscuous GDDIs highly. All the 194,752 putative DDIs were ranked as described in the text, and the ranks of GDDIs were extracted to create the plots. Promiscuity of a domain pair (*a*, *b*) = [N(*a*)+N(*b*)]/2 where N(*d*) is the number of times domain *d* occurs in a protein set. The concept lattices which are not attribute-reduced (**OA** and **oA**) *no longer exhibit the desired negative relationship* (Figure 11). Instead, they tend to rank promiscuous GDDIs less highly. The relationship is still strongly positive when the **Oa** rankings are used. **Oa** results are identical to the Associative method which is known to penalize promiscuous domain-pairs. There is still also a tendency for the **oa** concept lattice to rank promiscuous GDDIs less highly, but this positive relationship is not so apparent because scoreless GDDIs are included in the plot (they start at rank 28,457 and onwards to the right of the plot). When the **oa** concept lattice is used, only 59 of the 214 GDDIs have CB scores; the remaining GDDIs are scoreless and are ranked randomly but below the GDDIs with CB scores.

The anti-correlation in Figure 11 is observed with evaluation scenarios **B** and **C** (Figures S10 and S11 in File S1), which implies the robustness of the GDDI rank vs. promiscuity relationship against changes in PPIs. However, this relationship is vulnerable to changes in protein domain architecture. When the domains are shuffled (scenario **D**), both **OA** and **oA** GDDI rankings become positively related with promiscuity, i.e. less promiscuous GDDIs are ranked more highly (Figure 12). Domain shuffling destroys proteins with mixed architecture (Figure 10). Hence, mixed architecture proteins influences the ability of non-attribute-reduced concept lattices to rank promiscuous GDDIs highly.

### Domain Shuffling and Mixed Architecture Proteins

**Figure 13:**
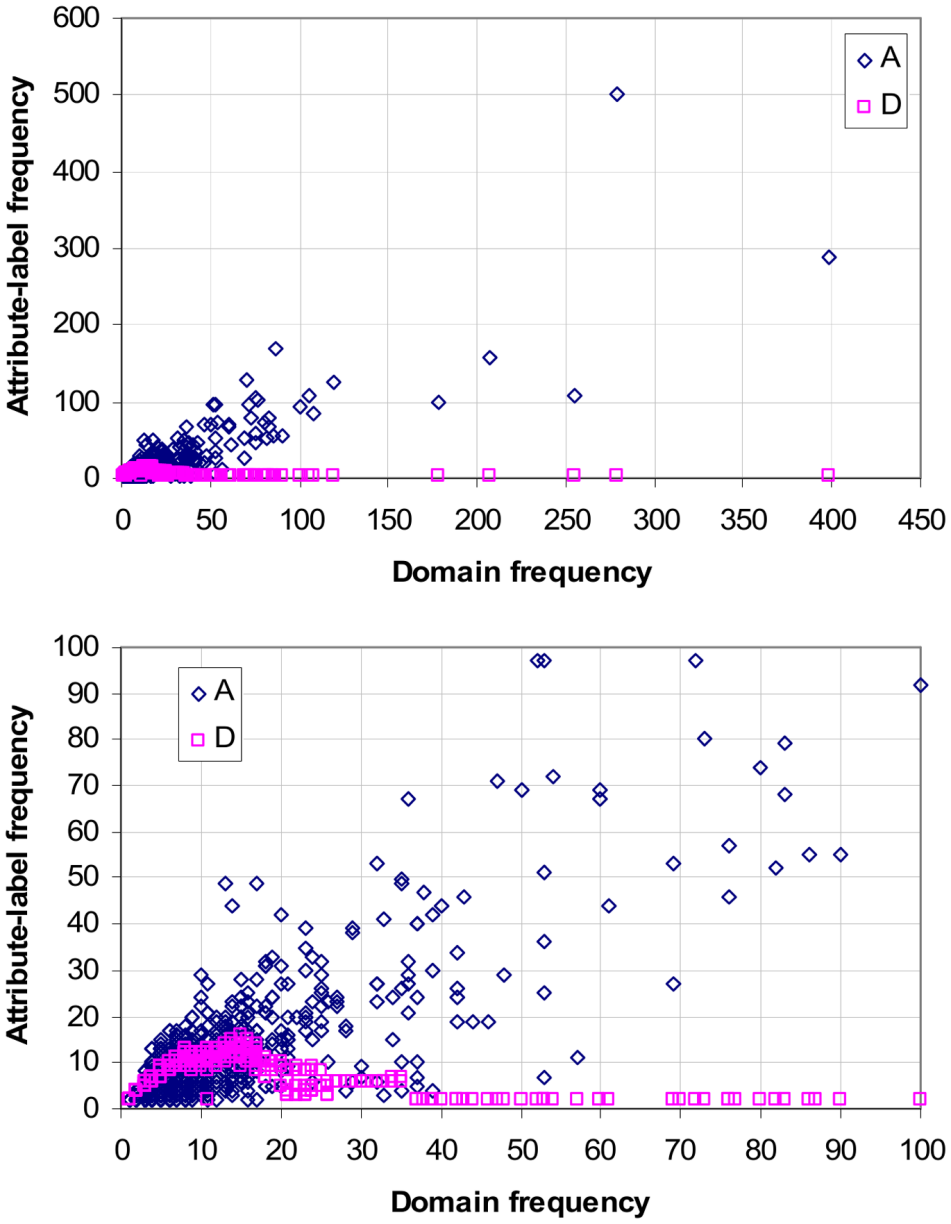
Effect of domain-shuffling on the relation between attribute-label frequency and domain frequency. The scatter-plot at the bottom zooms in on the first 100 domain frequency values. There is a strong positive correlation prior to domain-shuffling (scenario **A**) which is lost after domain-shuffling (scenario **D**). The lost of this strong positive correlation impairs piggy-backing (Figure 14).

Domain shuffling in scenario **D** destroys the mixed architecture proteins (Figure 10) and changes the protein-domain context into a more fragmented one. The number of concepts increases from 8,892 to 11,032, and the length of the longest path from the top element to the bottom element of the concept lattice reduces from 10 to 4. The most pertinent consequence of the changes in the concept lattice in **D** is that there is no longer a strong positive correlation between attribute-label frequency (the number of times a domain appears in an attribute-label set in a concept lattice) and domain frequency (the number of times a domain appears in a protein in a set of input proteins) (Figure 13). Instead, attribute-labels appear almost uniformly in frequency regardless of domain frequency, which is a similar relationship that attribute-labels have with domain frequency in attribute-reduced concept lattices. It is not surprising then that both **oA** and **OA** produce similar results to **Oa** in Figure 12, i.e. rank more promiscuous domain-pairs less highly.

**Figure 14:**
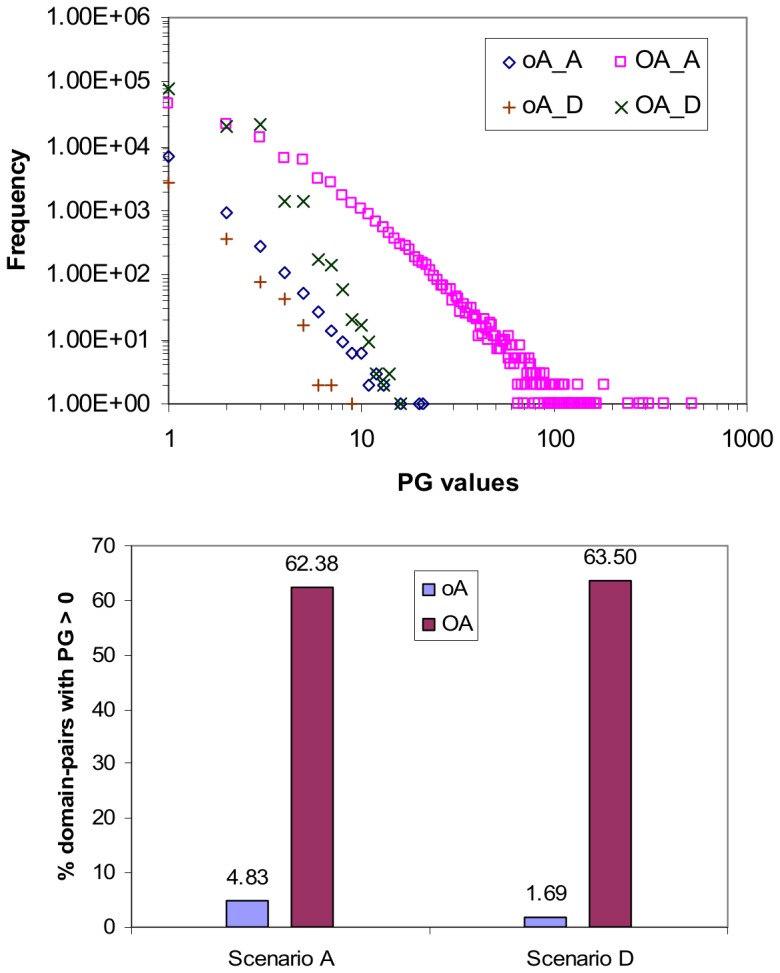
Effect of domain-shuffling on PG values and piggy-backing. In both scenarios **A** and **D**, **OA** produces a larger range of PG values than **oA** (top). Prior to domain-shuffling (**A**), the range of PG values is [0, 523] for **OA** and [0,21] for **oA**. Post domain-shuffling (**D**), the range of PG values is [0, 16] for **OA** and [0, 9] for **oA**. A reason for this is **OA** uses many more concepts than **oA** to score domain-pairs (Table S4 in File S1). In both scenarios **A** and **D**, **OA** has more piggy-backing, as evidenced by its much higher proportion of domain-pairs with PG >0. A domain-pair with PG >0 means its CB score is the result of one or more piggy-backs. The text discusses the pros and cons of **OA**’s higher piggy-backing potential. For **oA**, the proportion of domain-pairs with PG >0 drops from 4.83% (8555/177233) to 1.69% (3284/194752) when the domains are shuffled. With fewer piggy-backs, the results for **oA** deteriorate. PG is always 0 for attribute-reduced concept lattices (**oa** and **Oa**).

The lost of the strong positive correlation between attribute-label frequency and domain frequency means a reduction in the number of contexts (sets of protein-pairs) to evaluate promiscuous domain-pairs, and a decrease in piggy-backing opportunities. Figure 14 (top) shows the decrease in the range of PG values as a result of domain shuffling. The decrease in piggy-backing opportunities becomes more severe when the concept lattice is object-reduced. Under scenario **D**, only 1.69% of the domain-pairs evaluated with **oA** have PG >0 (Figure 14 bottom). While the range of PG values becomes much narrower for **OA** under scenario **D**, the proportion of domain-pairs still able to piggy-back in **OA** remains above 62%. With a weakened piggy-backing mechanism, both **oA** and **OA** no longer rank more promiscuous domain-pairs more highly (Figure 12).

### GDDI Recovery

GDDI recovery is concerned with finding as many GDDIs as possible while making as few mistakes as possible. The GDDIs are sourced from the iPfam database [6]. This is a test of quantity, not quality. All GDDIs are treated equally, i.e. GDDIs are not differentiated by their promiscuity. GDDI recovery is performed by inspecting domain-pairs in descending rank order and obtaining the True Positive Rate (TPR) and False Positive Rate (FPR) for each unique <CB, PG> score [17]. TPR is the number of GDDIs found so far divided by the total number of GDDIs to find. FPR is the number of non-GDDIs met so far divided by the total number of non-GDDIs. The total number of non-GDDIs is the total number of DDIs less the total number of GDDIs.

For the problem of identifying *reliable* DDIs, it is preferable to keep the FPR low. Even at FPR = 0.2, there are already 35,290 false positives to eliminate either experimentally or through literature search in scenario **A**. Further, errors in predicting DDIs can be propagated to other areas that rely on DDI data, such as prediction of protein-protein interactions and identification of protein binding surfaces. Hence, the argument for low FPR or high Specificity. A cost-efficient high-throughput method to eliminate non-interacting domain-pairs with high confidence would render computational efforts to predict reliable DDIs obsolete.

**Figure 15:**
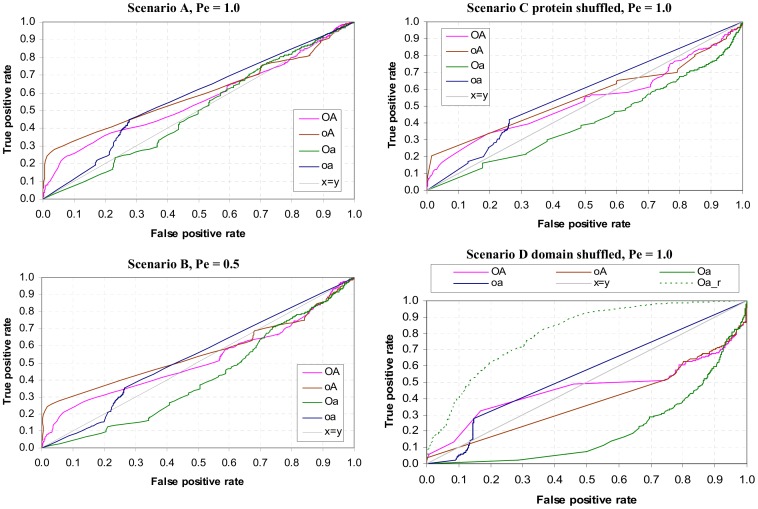
Recovery of GDDIs [6]. GDDIs make up 0.44% of DDIs in **A**, 0.60% in **B**, 0.21% in **C** and 0.11% in **D**. Median promiscuity of GDDIs to DDIs is 7.0∶5.0 in **A**, 8.5∶5.5 in **B**, 9.5∶5.5 in **C** and 15.0∶4.0 in **D**. Except for the concocted scenario **D**, at high Specificity (FPR ≤ 0.2), concept-based rankings produced with concept lattices that are not attribute-reduced (**OA** and **oA**) outperform (have higher Sensitivity or larger TPR) those by concept lattices that are attribute-reduced (**Oa** and **oa**). In scenarios **A** to **C**, **oA** outperforms **OA**. This outcome holds even when DOMINE [18] and 3did [19] domain-pairs are used to evaluate the rankings (Figure 16). The quality of the **oA** domain-pair rankings becomes more evident by examining its top 100 domain-pairs (Figure 17 and File S2). **OA**’s poorer performance in scenarios **A** to **C** is attributed to “excessive” piggy-backing. Scenario **D** shows that **oA**’s GDDI recovery is more sensitive to changes in protein domain architecture than **OA**’s. The changes introduced by domain shuffling in **D** reduce **oA**’s piggy-backing potential and its GDDI recovery suffers as a result. In contrast, **OA** is able to retain some of its previously “excessive” piggy-backing potential (Figure 14). Nonetheless, the GDDIs recovered at low FPR by **OA** in scenario **D** are of the less promiscuous variety (Figure 12). In all four scenarios, the **oa** concept lattice leaves at least 72% domain-pairs and at least 55.30% GDDIs scoreless. Also, the **Oa** rankings produced the worst GDDI recovery performance in all four scenarios.

The TPR vs. FPR or ROC graphs are shown in Figure 15. Except for the concocted scenario **D**, when FPR ≤ 0.2, rankings produced by the non-attribute-reduced concept lattices (**OA** and **oA**) have larger TPR or higher Sensitivity than the attribute-reduced concept lattices (**Oa** and **oa**). This outcome supports the hypothesis that using concept lattices that are not attribute-reduced is necessary to create a favourable condition for the concept-based scoring method to identify GDDIs. This outcome is robust to changes in the PPI data as evidenced by the results from scenarios **B** and **C**. The **oa** rankings become slightly more competitive when FPR ≥0.25, but the rankings quickly become random as **oa** is unable to score many domain-pairs due to empty object-label and empty attribute-label sets in the **oa** concept lattice. For instance, 72.14% of the possible domain-pairs and 55.3% of GDDIs are left scoreless by **oa** in scenario **A**. The **Oa** rankings or Associative method gave the worst GDDI recovery performance.

Except for the concocted scenario **D**, up to an FPR as large as 0.5, **oA**’s GDDI recovery dominates that of **OA**. A closer look at the top 100 domain-pairs (File S2) reveals that this could be due to excessive piggy-backing in the **OA** concept lattice. With no empty object-label sets, **OA** generates many more contexts than **oA** to evaluate domain-pairs thereby creating more piggy-backing opportunities. A consequence of this is more opportunity in **OA** to promote promiscuous domain-pairs including non-GDDIs up the ranks. Figure 14 shows the distribution of PG values generated in scenario **A**. The **OA** PG values span a larger range than the **oA** PG values. 62.4% of the domain-pairs have PG >0 when the **OA** concept lattice is used, while only 4.8% do with **oA**. **OA**’s more diverse PG values help it achieve a higher pass rate in the Nye test than **oA** (Results section). However, high ranking non-GDDIs do at times interfere with **OA**’s Nye test results (Results section).

**Figure 16:**
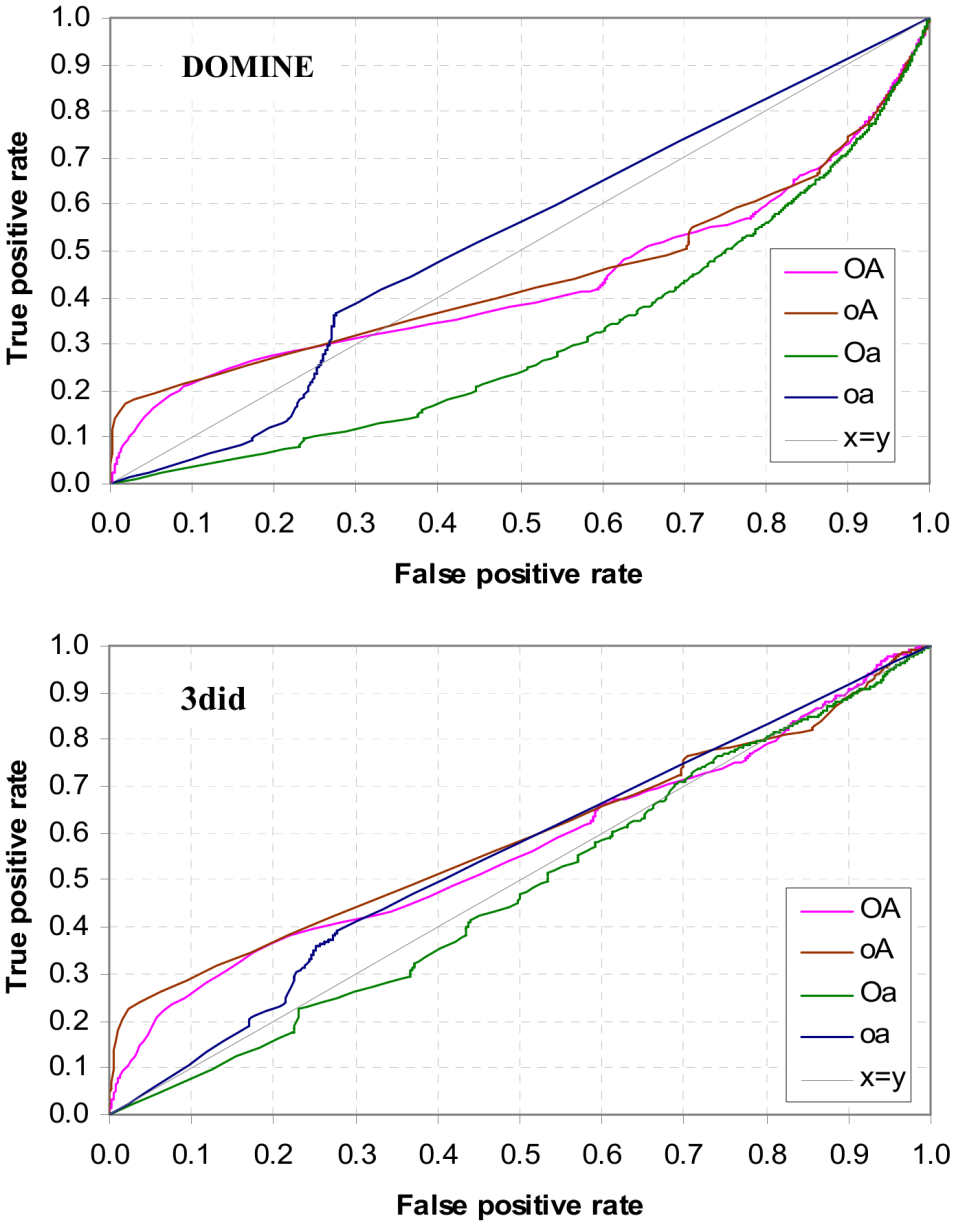
Recovery of domain-pairs in DOMINE [18] (top), and in 3did (Jul-25-2013 release) [19] (bottom). At high Specificity (FPR ≤ 0.2), **oA**’s TPR dominates the other three rankings. All rankings were made under scenario **A** conditions.

**Figure 17:**
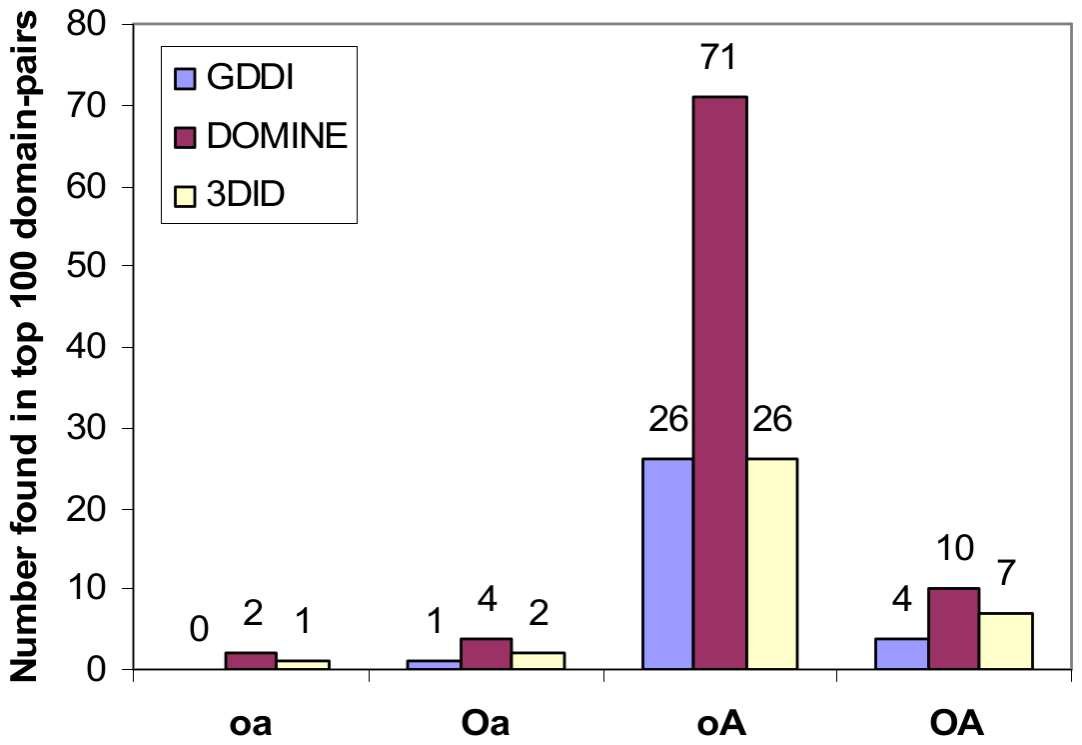
Number of GDDIs [6], DOMINE domain-pairs [18] and 3did domain-pairs [19] found in the top 100 domain-pairs. Of the four rankings (all made under scenario A conditions), **oA** contains the most number of GDDIs, DOMINE domain-pairs and 3did domain-pairs in the top 100 domain-pairs (File S2). The 26 GDDIs for **oA** intersect with, but is not the same set as, the 26 3did domain-pairs for **oA**. Amongst **oA**’s top 100 domain-pairs is domain-pair (TPR, WD40) which is not a GDDI and whose interaction is neither recorded in DOMINE nor predicted by 3did. However, a possible 3D structure for the obesity-related protein adipose (adp) involves interaction between TPR and WD40 domains [20].

Further evidence that the **oA** domain-pair rankings dominates that of **OA** is given in Figure 16, which shows the recovery of domain-pairs in DOMINE [18] and in 3did [19]. DOMINE is a collection of known and predicted domain-pairs harvested from experiments with high-resolution 3D structures and the results of 15 computational methods. 7,766 of the 177,233 domain-pairs in the Riley dataset are found in DOMINE. 3did is a catalog of domain-based interactions computationally derived from Pfam domain definitions and PDB 3D structures. 1,148 of the 177,233 domain-pairs in the Riley dataset are found in 3did (Jul-25-2013 release). Of the 7,766 DOMINE domain-pairs, 663 are GDDIs and 951 are 3did domain-pairs. Of the 1,148 3did domain-pairs, 650 are GDDIs. There are 581 domain-pairs in the Riley dataset which is common to all GDDIs, DOMINE and 3did. At high Specificity (FPR ≤ 0.2), **oA**’s TPR dominates the other three rankings. Of the four rankings (all made under scenario A conditions), **oA** contains the most number of GDDIs, DOMINE domain-pairs and 3did domain-pairs in the top 100 domain-pairs (Figure 17). Amongst **oA**’s top 100 domain-pairs is domain-pair (TPR, WD40) which is not a GDDI and whose interaction is neither recorded in DOMINE nor predicted by 3did. However, a possible 3D structure for the obesity-related protein adipose (adp) involves interaction between TPR and WD40 domains [20].

The discussion so far all point to **oA** producing better GDDI recovery results than **OA**. However, **oA** is more vulnerable to changes in protein domain architecture than **OA**. When the domains are shuffled to reduce the number of mixed architecture proteins (scenario **D**), **oA**’s TPR which previously has been above 0.2 when FPR ≤ 0.2 drops to less than 0.2. This decline in **oA**’s GDDI recovery performance supports the notion that mixed architecture proteins play an important role in the success of concept-based scoring.

Domain shuffling also affects **OA**’s prioritization of promiscuous domain-pairs, but the effect on GDDI recovery is dampened by **OA**’s ability to provide many more contexts to evaluate domain-pairs. In scenario **D**, the **oA** rankings are produced with only 53.71% of the concepts. In contrast, **oA** uses 82.38% of the concepts in the other three scenarios (Table S3 in File S1). **OA** always uses 100% of the concepts (with the top and bottom elements are excluded). While domain shuffling reduces the range of PG values for **OA**, the proportion of domain-pairs still able to piggy-back in **OA** remains above 62%. In contrast, only 1.69% of the domain-pairs evaluated with **oA** have PG >0 (Figure 14 bottom). Thus even in concocted scenario **D**, the ability to piggy-back exerts a strong influence on the performance of concept-based ranking.

As with the other three scenarios, many domain-pairs (85.39% of the possible domain-pairs and 72.43% of GDDIs) are left scoreless by **oa** in scenario D. The dismal performance in **Oa**’s GDDI recovery is emphasized by the fact that reversing **Oa**’s ranking substantially improves GDDI recovery (Oa_r plot in Figure 15). There is no potential for piggy-backing when an attribute-reduced concept lattice is used.

### The Nye Test

This test is concerned with whether *the* highest ranking DDI for a GPPI is a GDDI. It was first performed by Nye *et al*. [15] to predict domain-domain contacts for interacting protein pairs, and was used in [6] to evaluate their DDI prediction method. Strict comparison is used for the Nye test in this paper, i.e. if a GPPI has a non-GDDI with the same highest rank as a GDDI, the test fails for said GPPI. A GPPI with more DDIs and a smaller GDDI/DDI ratio is more challenging for the Nye test. A demonstration of how to conduct the Nye test can be found in Figure S12 in File S1.

**Figure 18:**
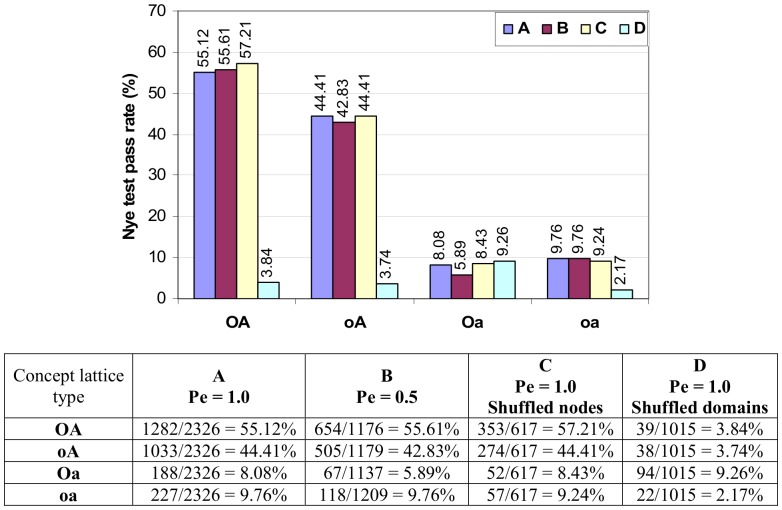
Nye test pass rate for the four concept lattice types in the four test scenarios. A higher pass rate means a larger proportion of GPPIs have a GDDI as the highest ranking DDI. Except for **D**, concept lattices which are not attribute-reduced (**oA** and **OA**) have significantly higher pass rates than concept lattices that are attribute-reduced. The number of GPPIs is different in each scenario since GPPIs depend on GDDIs and PPIs, both of which are affected in scenarios **B** to **D** (Table S3 in File S1). GPPIs with only one DDI are included in the counts. The 2,326 GPPIs in **A** includes 546 single-DDI GPPIs. The Nye test results supports the hypothesis that in addition to a concept lattice that is not attribute reduced, mixed architecture proteins are also necessary to create favourable conditions for concept-based scoring to do well.

Results of the Nye test on the Riley dataset are summarized in Figure 18. A higher pass rate means a larger proportion of GPPIs have a GDDI as the highest ranking DDI. The concept lattices that are not attribute-reduced (**OA** and **oA**) produce much higher pass rates than the attribute-reduced concept lattices (**Oa** and **oa**). OA’s higher pass rate is attributed to its greater piggy-backing potential (Figure 14). This trend is consistent across all scenarios except **D** where **Oa** performs slightly better than the other three and appears less affected by changes in protein architecture. **Oa**’s result is identical to that of the Associative Method and strictly speaking is not concept-based.

The large drop in **OA**’s and **oA**’s Nye test pass rates in scenario **D** reinforce the importance of the ability to piggy-back for the success of the concept-based scoring method, and this ability is substantially enhanced by the strong presence of mixed architecture proteins. As previously discussed, domain shuffling changes the domain architecture of proteins, specifically mixed architecture proteins are now conspicuously absent in the protein population. This architectural change affects the shape of the concept lattice and a pertinent consequence is the lost of the strong positive correlation between domain frequency and attribute-label frequency (Figure 13). This lost reduces the number of contexts (concept-pairs) within which to evaluate domain-pairs, and as a result piggy-backing is drastically reduced (Figure 14). The Nye test results supports the hypothesis that in addition to a concept lattice that is not attribute-reduced, mixed architecture proteins are also necessary to create favourable conditions for concept-based scoring to do well.

**Figure 19:**
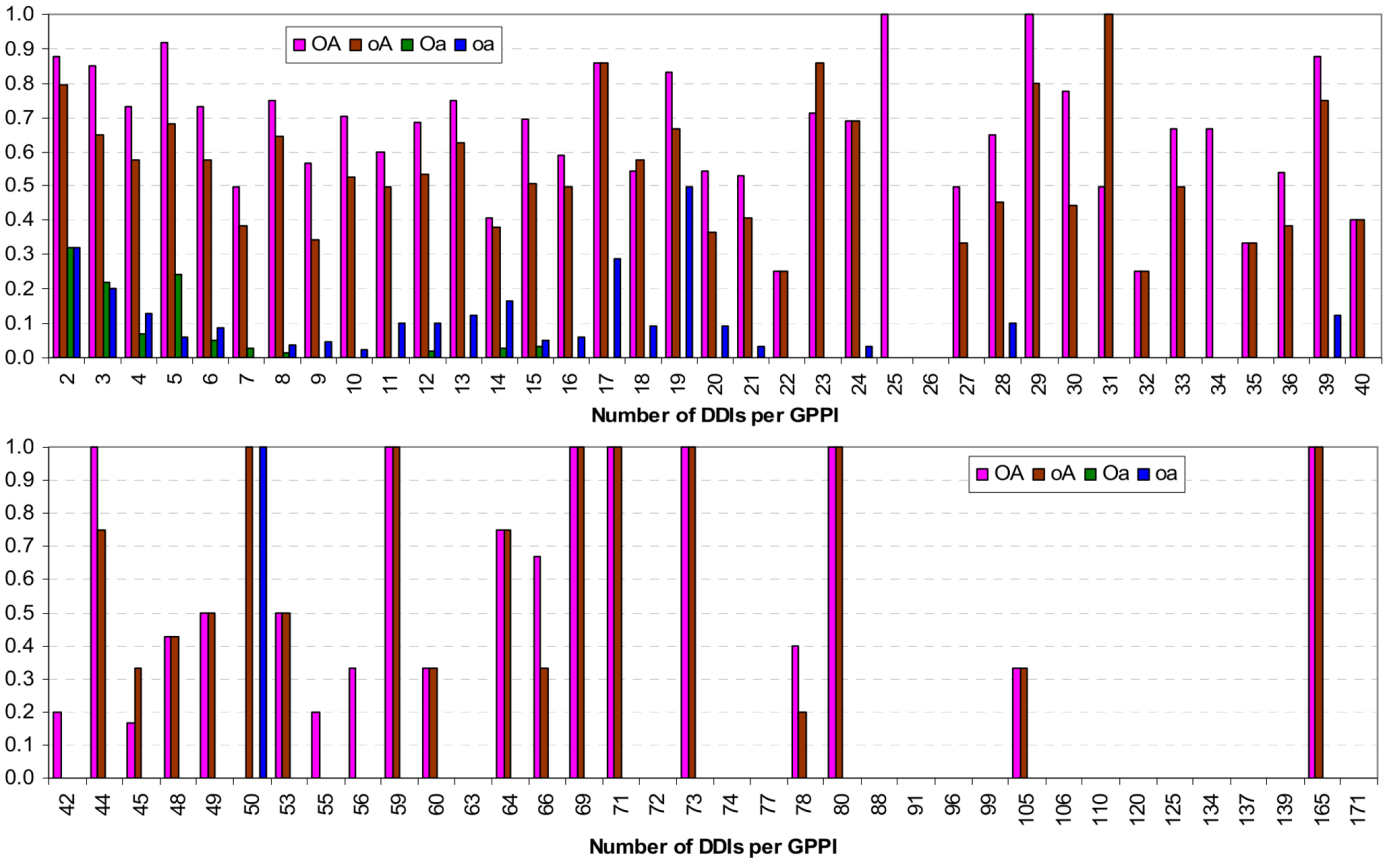
Nye test results by difficulty. The Nye test is more difficult as the number of DDIs per GPPI increases (Figure S13 in File S1). Each bar shows the fraction of GPPIs with *x* number of DDIs that pass the Nye test, i.e. where a GDDI is the highest ranking DDI, in scenario **A**. As the level of difficulty increases, concept-based scoring and ranking made with the attribute-reduced concept lattices (**Oa** and **oa**) become less able to pass the Nye test. With a few exceptions, regardless of difficulty, more GPPIs pass the Nye test with the **OA** ranking than with the **oA** ranking. This is attributable to **OA**’s greater potential for piggy-backing (Figure 14). However, the piggy-backing mechanism is also available to promiscuous non-GDDIs, and this causes some GPPIs to fail the Nye test when using the **OA** ranking. For example, the GPPI with 50 DDIs is (2252, 2530). This GPPI is supported by only one GDDI (AAA, AAA), which has a promiscuity of 100. **oA** ranks (AAA, AAA) as the highest domain-pair for GPPI (2252, 2530) and so the Nye test is passed. **OA** ranks (AAA, AAA) as the second highest, below (Pfam-B_1, AAA) which has a promiscuity of 189.5 but is not a GDDI, and so the Nye test is failed. The Nye test is also passed by **oa** since (AAA, AAA) is the only domain-pair with a score for GPPI (2252, 2530). However, **oa**’s Nye test performance on a GPPI with this high level of difficulty is more the exception than the norm.

The Nye test results for scenario A is broken down by difficulty in Figure 19. The Nye test is more difficult for GPPIs that generate a larger number of DDIs. Most GPPIs generate a small number of DDIs, but there are GPPIs in the Riley dataset that generate well over 40 DDIs each (Figure S13 in File S1). As the level of difficulty increases, concept-based scoring and ranking made with the attribute-reduced concept lattices (**Oa** and **oa**) become less able to pass the Nye test. None of the GPPIs that generate more than 50 DDIs pass the Nye test when **oa**’s ranking is used. For the **Oa** concept lattice (the Associative method), the cut-off point is even earlier, at 16 DDIs. In contrast, several GPPIs with more than 50 DDIs could still pass the Nye test when ranking is based on concept-based scores computed with the non-attributed-reduced concept lattices (**OA** and **oA**).

With a few exceptions, regardless of difficulty, more GPPIs pass the Nye test with the **OA** ranking than with the **oA** ranking (Figure 19). This is attributable to **OA**’s greater potential for piggy-backing. Compared with **oA**, **OA** produced a larger range of PG values and a greater proportion of its domain-pairs piggy-backed (Figure 14). However, the piggy-backing mechanism is also available to promiscuous non-GDDIs, and this causes some GPPIs to fail the Nye test when using the **OA** ranking. For example, the GPPI with 50 DDIs is (2252, 2530). This GPPI is supported by only one GDDI, (AAA, AAA), which has a promiscuity of 100. **oA** ranks (AAA, AAA) as the highest domain-pair for GPPI (2252, 2530) and so the Nye test is passed. **OA** ranks (AAA, AAA) as the second highest, below (Pfam-B_1, AAA) which has a promiscuity of 189.5 but is not a GDDI, and so the Nye test is failed. The Nye test is also passed by **oa** since (AAA, AAA) is the only domain-pair with a score for GPPI (2252, 2530). However, **oa**’s Nye test performance on a GPPI with this high level of difficulty is more the exception than the norm.

## Discussion

**Figure 20:**
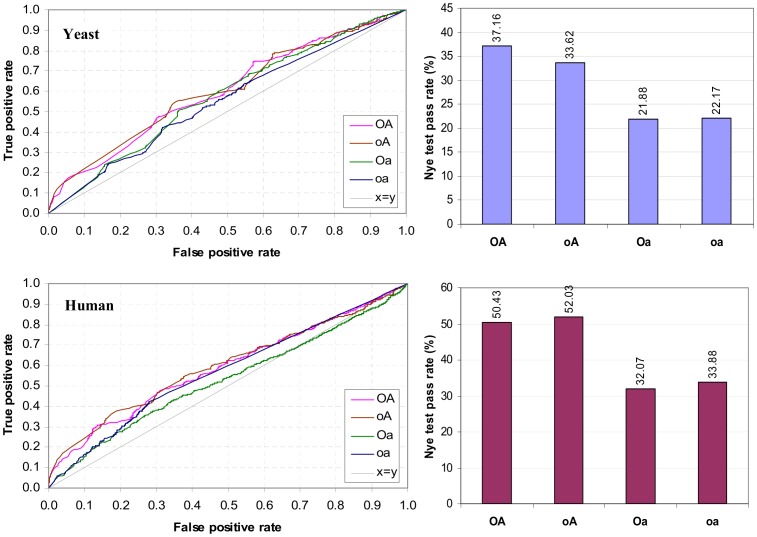
Concept-based scoring and ranking in the absence of Pfam-B domains. Rankings were made under scenario **A** conditions. Recovery of 3did domain-pairs for yeast (top-left) and human (bottom-left). Without Pfam-B domains, the ROC curve for **Oa** is no longer below the y = x line as it was in Figure 15. Nonetheless, at high Specificity (FPR ≤ 0.2), the Sensitivity (TPR) of non-attribute-reduced concept lattices (**OA** and **oA**) still dominate that of the attribute-reduced concept lattices (**Oa** and **oa**) although now it is no longer as clear as it was in Figure 15 that **oA**’s TPR dominates **OA**’s TPR. The point is not to quibble about the difference between **OA** and **oA**, but between attribute-reduced where piggy-backs are impossible and non-attribute-reduced where piggy-backs are possible. The ROC curves show that in the absence of Pfam-B domains and on a more current dataset for single organisms, both **OA** and **oA** still outperform both **Oa** and **oa**. The Nye test for yeast (top-right) and for human (bottom-right) is also more successfully passed by both **OA** and **oA** than either **Oa** or **oa**.

**Figure 21:**
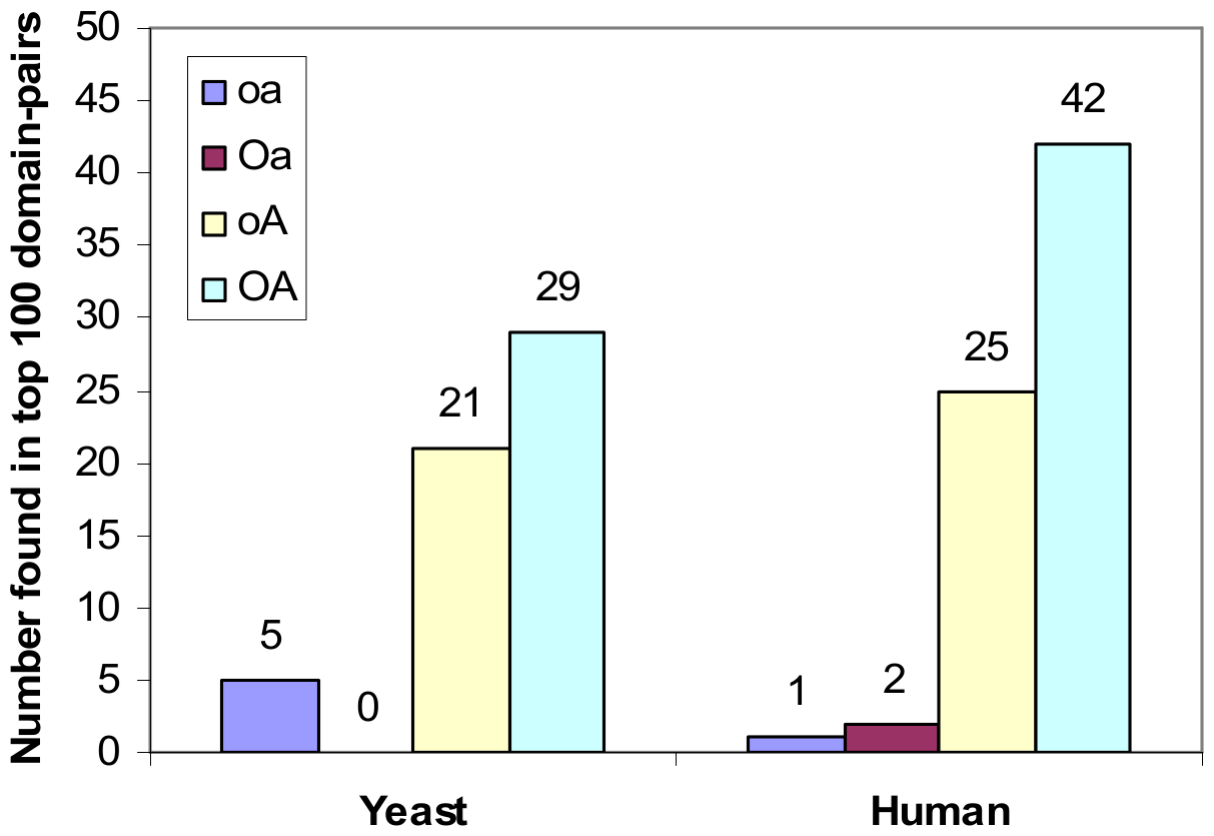
Number of 3did domain-pairs [19] found in the top 100 domain-pairs for Yeast and for Human. Without Pfam-B domains, both **oA** and **OA** still have more 3did domain-pairs in their top 100 than both **Oa** and **oa**. However, in contrast to Figure 17, now **OA** has more 3did domain-pairs than **oA**. This is because there are no promiscuous Pfam-B domains to cloud **OA**’s ranking.

The previous case with GPPI (2252, 2530) illustrates how the presence of promiscuous Pfam-B domains can cloud the Nye test results for **OA**. These promiscuous Pfam-B domains also suppress GDDI recovery for **OA**. All domain-pairs in **OA**’s top 100 that do not involve a Pfam-B domain is either a GDDI, or identified in DOMINE or 3did (File S2). However Pfam-B domains are more likely to be rare domains. Of the 12,455 domains in the Riley dataset, 9,720 (78%) are Pfam-B domains. Of the Pfam-B domains, 65% occur in only one protein and 93.5% occur in three or fewer proteins. As previously acknowledged [10], the presence of numerous rare Pfam-B domains suppresses the results for the Association method (**Oa**). Therefore, there is a need to evaluate the effectiveness of the concept-based scoring method in the absence of Pfam-B domains and on a more current dataset for single organisms. The organisms tested here are yeast (NCBI taxid 559292) and human (NCBI taxid 9606), and the data come from Biogrid (version 3.2.107) [22], Pfam (version 27.0) [21] and 3did (Jul-25-2013 release) [19] which are all publicly available and the most recent at the time of preparing this publication (Table S5 in File S1). The results are reported in Figures 20 and 21. All rankings were made under scenario **A** conditions.

Without Pfam-B domains, the ROC curve for **Oa** is no longer below the y = x line as it was in Figure 15. Nonetheless, at high Specificity (FPR ≤ 0.2), the Sensitivity (TPR) of non-attribute-reduced concept lattices (**OA** and **oA**) still dominate that of the attribute-reduced concept lattices (**Oa** and **oa**) although now it is no longer as clear as it was in Figure 15 that **oA**’s TPR dominates **OA**’s TPR (Figure 20 left). Without Pfam-B domains, both **oA** and **OA** still have more 3did domain-pairs in their top 100 than both **Oa** and **oa** (Figure 21). However, in contrast to Figure 17, now **OA** has more 3did domain-pairs than **oA**. This is because there are no promiscuous Pfam-B domains to cloud **OA**’s ranking. The point here is not to quibble about the difference between **OA** and **oA** (although computation wise, **OA** takes longer to complete than **oA**), but between attribute-reduced where piggy-backs are impossible and non-attribute-reduced where piggy-backs are possible. The results in Figures 20 and 21 show that in the absence of Pfam-B domains and on a more current dataset for single organisms, both **OA** and **oA** can still outperform both **Oa** and **oa**. The Nye test for yeast and for human is also more successfully passed by both **OA** and **oA** than either **Oa** or **oa** (Figure 20 right).

It may appear from these results that Pfam-B domains should be excluded since any domain-pair involving a Pfam-B domain is not documented in any current database as a reliable DDI. However, Pfam-B domains play a role, through the mixed architecture proteins, in increasing the number of contexts within which to evaluate domain-pairs and thus enhance piggy-backing potential. A suggestion for future work is to expand the attribute set beyond domains to for example combination of domains in the form of bi-grams [2] or supra-domains [10], or to include sequence motifs for a richer characterization of proteins.

To conclude, a method based on Formal Concept Analysis [9] to infer reliable domain-domain interactions from protein-protein interactions was proposed and shown to be feasible in the presence of domain promiscuity. The effectiveness of the proposed method is due to a piggy-backing mechanism which is made possible in concept lattices that are not attribute-reduced, and enhanced by mixed architecture proteins. The problem of using highly reliable domain-pairs to predict protein-protein interactions with high accuracy remains a future challenge.

## Supporting Information

File S1 (PDF)

File S2 (XLS)
