# Supplementary material for: Inferring Domain-Domain Interactions from Protein-Protein Interactions with Formal Concept Analysis
Source: PLoS One. 2014 Feb 19;9(2):e88943. doi: 10.1371/journal.pone.0088943 (PMC3929762; doi:10.1371/journal.pone.0088943)
Supplement: File S1 — (PDF) [file pone.0088943.s001.pdf]

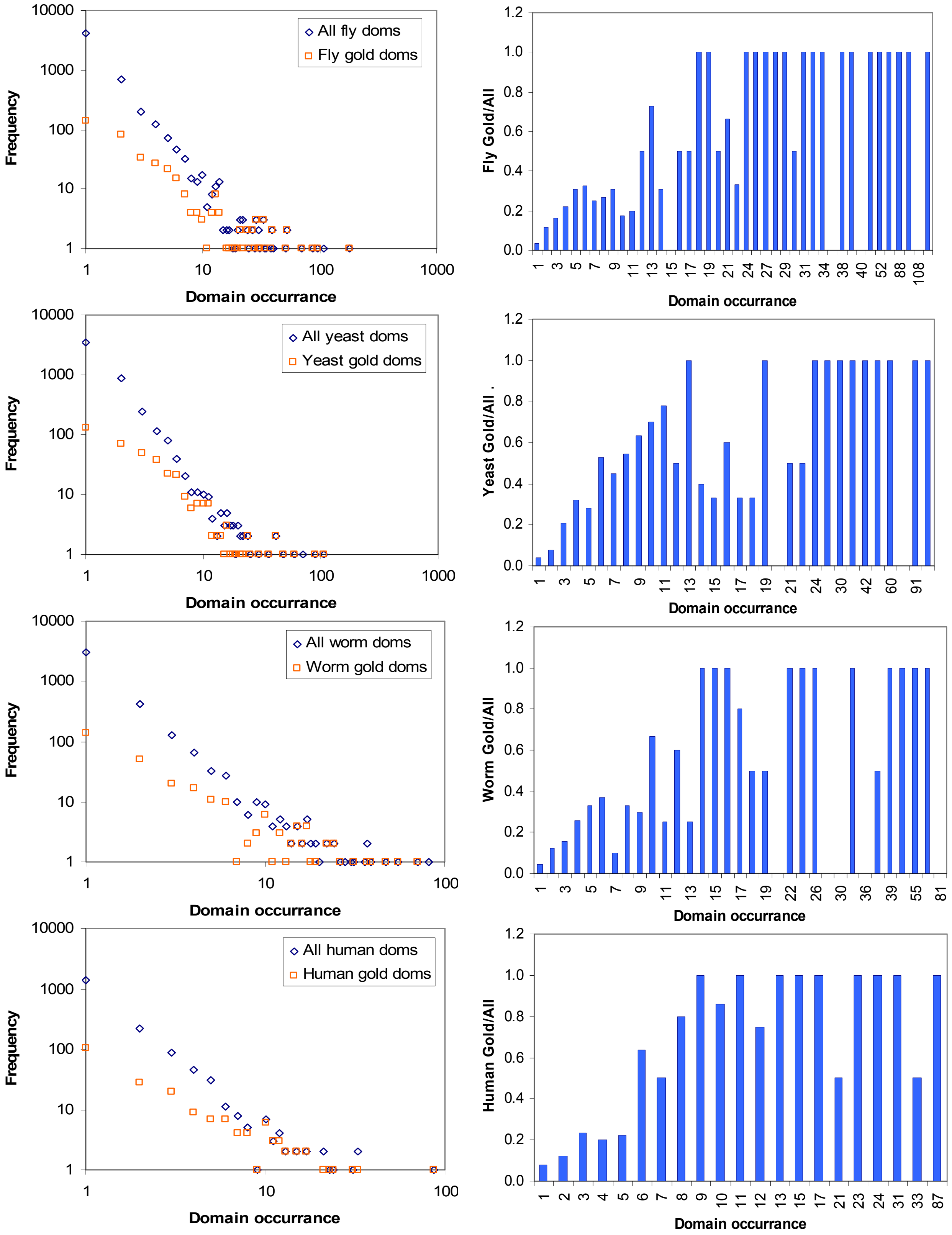

Figure S1 (Left) The log-log plots show for each organism that rare domains occur less frequently in the set of gold domains than in the set of all domains. (Right) In general, for each organism the more frequently a domain occurs, the more likely it is to be a gold domain.

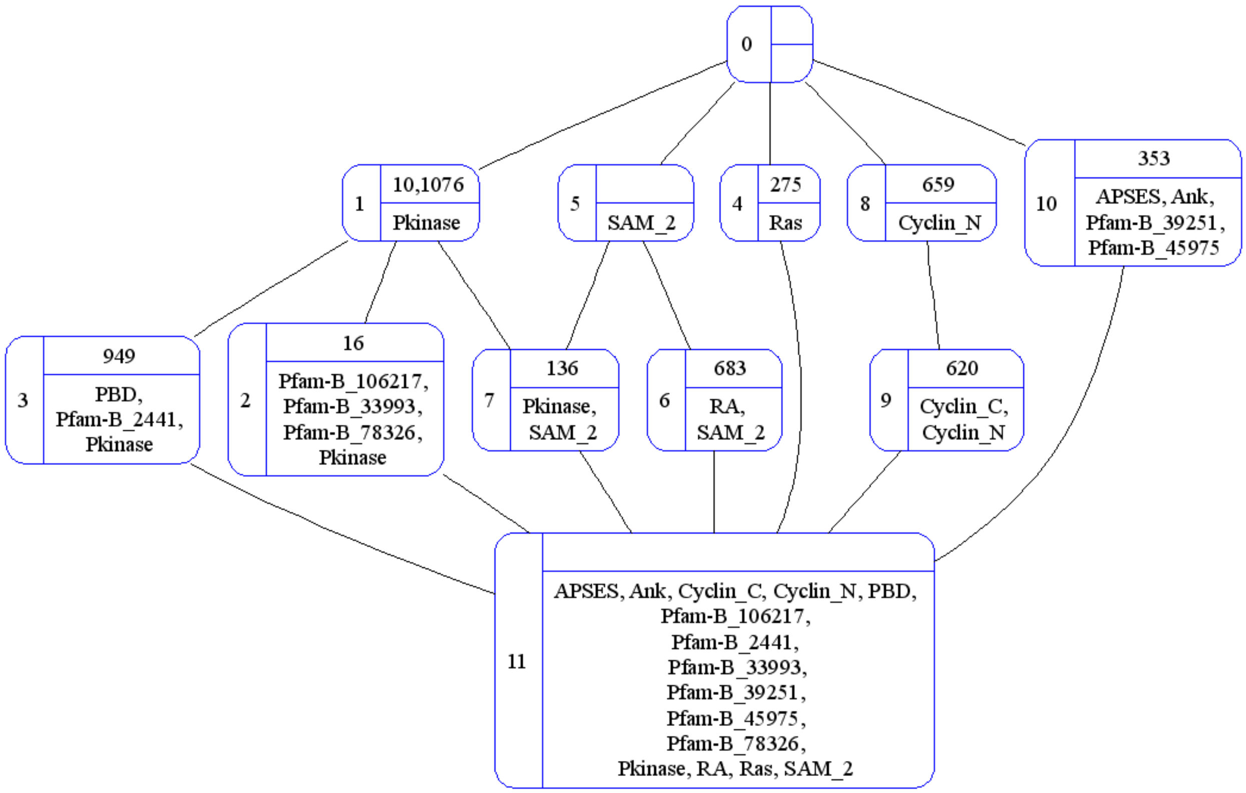

Figure S2 The **oA** concept lattice for the *S. pombe* context in Table 2.

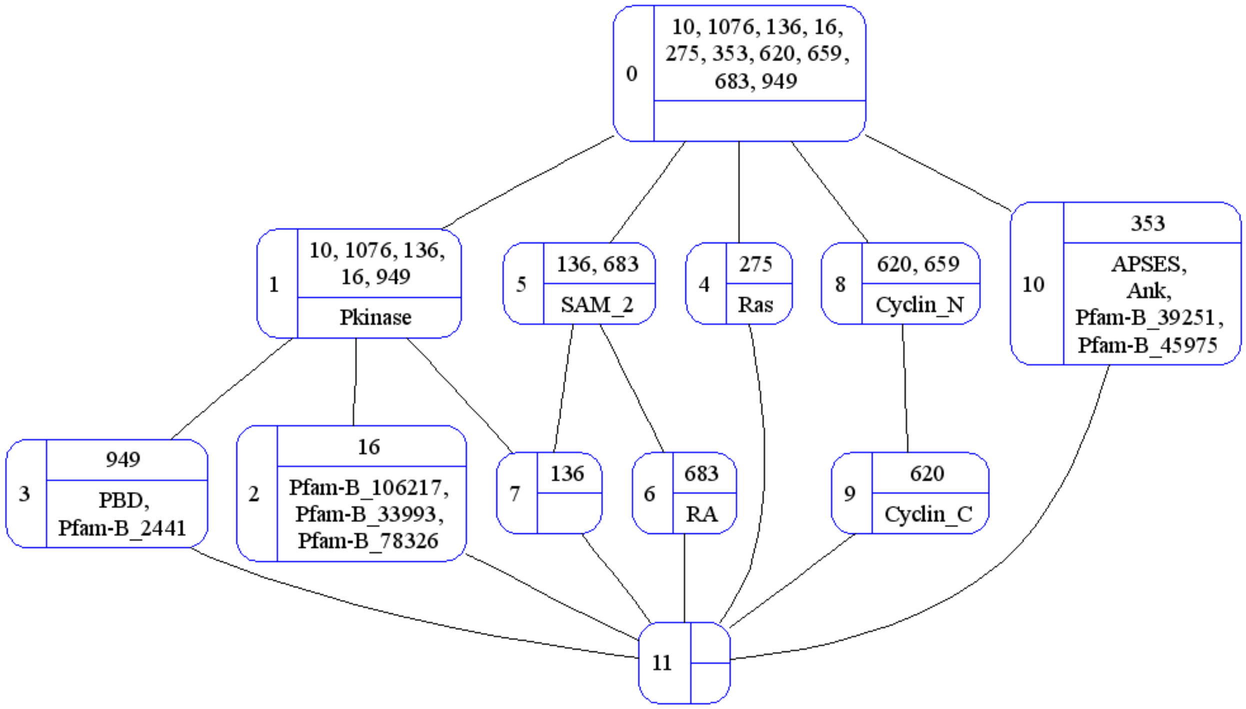

Figure S3 The **Oa** concept lattice for the *S. pombe* context in Table 2.

Table S1 Cross-table for the toy example in Fig. 1 [8].

		Attributes							Number of attributes per object
		Y	B	A	O	G	R	V	
Objects	0	x	x						2
	1		x	x	x	x	x		5
	2						x		1
	3				x		x		2
	4	x	x			x	x	x	5
	5				x		x		2
	6		x						1
	7		x						1
	8					x	x		2
	9						x		1
	10		x	x	x	x		x	5
	11						x		1
Attribute Frequency		2	6	2	4	4	8	2	

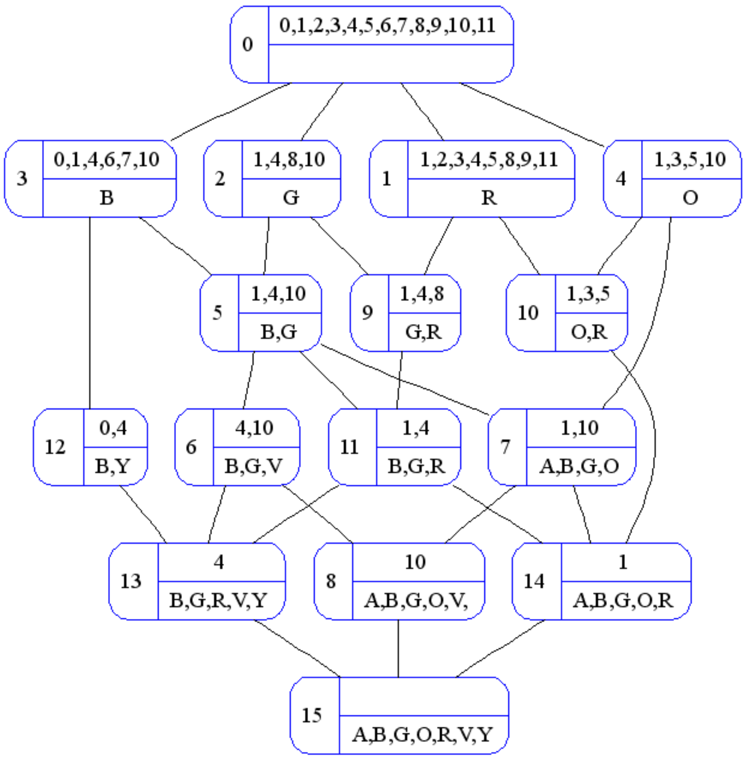

Figure S4 The fully-labeled (**OA**) concept lattice for the context in Table S1.

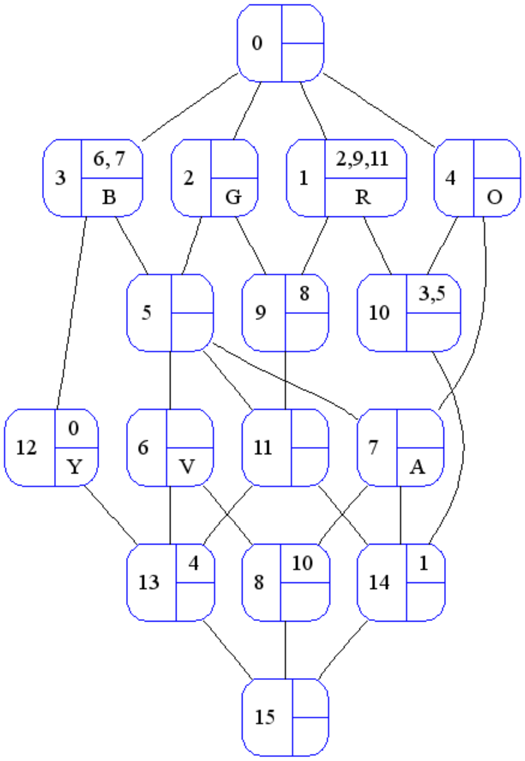

Figure S5 The reduced (**oa**) concept lattice for the context in Table S1.

Attributes with frequency > 4 (a.k.a. the promiscuous attributes: B, G, R and O) appear in concepts one step away from the top concept. The remaining attributes Y, V and A all appear only twice in the set of objects, and they appear at least two steps away from the top concept. Promiscuous attributes appear towards the top and rare attributes appear towards the bottom of an attribute-reduced concept lattice.

Objects comprising only one frequently occurring attributes (6, 7, 2, 9 and 11) appear in concepts one step away from the top concept. Objects with two or more promiscuous attributes only (0, 8, 3, 5) appear in concepts at least two steps away from the top concept. The objects comprising promiscuous and rare attributes (mixed attribute architecture: 4, 10, 1) appear one step away from the bottom concept. Objects comprising only promiscuous attributes appear towards the top of an object-reduced concept lattice. Mixed attribute objects appear towards the bottom of an object-reduced concept lattice.

The ten *S. pombe* proteins in the Riley dataset:

The presence (absence) of an edge between two nodes denotes an interaction (non-interaction). A bolded edge signifies that the two endpoint proteins form a GPPI. A GPPI is a protein-protein interaction that is supported by at least one gold standard domain-pair (GDDI).

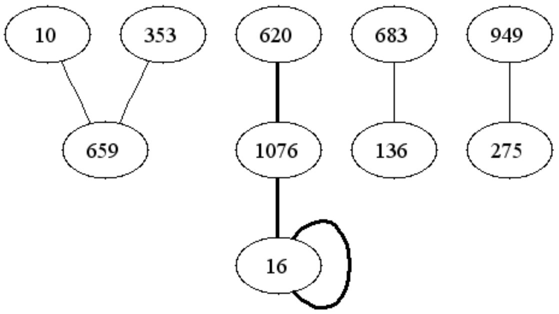

Concept-based scoring for GDDI(Pkinase, Pkinase) with *S. pombe* PPIs.

Average promiscuity of domain-pairs produced by $A^L(c_1) \times A^L(c_2)$	Concept-pairs (c_1, c_2) for GDDI(Pkinase, Pkinase)	**OA** Figure 6		**oA** Figure S2		**Oa** Figure S3		**oa** Figure 7	
		M	M+Z	M	M+Z	M	M+Z	M	M+Z
5.0	1 × 1	2	15	0	3	**2**	**15**	0	3
-	1 × 3	0	5	0	2	-	-	-	-
3.5	1 × 2	2	5	1	2	-	-	-	-
-	1 × 7	0	5	0	2	-	-	-	-
-	3 × 3	0	1	0	1	-	-	-	-
-	3 × 2	0	1	0	1	-	-	-	-
-	3 × 7	0	1	0	1	-	-	-	-
2.0	2 × 2	**1**	**1**	**1**	**1**	-	-	-	-
-	2 × 7	0	1	0	1	-	-	-	-
-	7 × 7	0	1	0	1	-	-	-	-
Concept-based score **CB** = $\max[\log_2(M/(M+Z))]$		$\log_2(1/1)$		$\log_2(1/1)$		$\log_2 (2/15)$		scoreless	
Number of score improvements (piggy-backs) **PG**		2		1		0		-1	

In **OA** (Figure 6) and **oA** (Supplementary Figure S2), Pkinase appears in the attribute-label set of concepts c1, c3, c2 and c7, which yields the 10 concept-pairs in the table. In **oa** (Figure 7) and **Oa** (Supplementary Figure S3), Pkinase appears in the attribute-label set of concept c1 only and yields a single concept-pair. Concept-based scoring works the same way regardless of concept lattice type. For demonstration purpose, take c1 × c2 under **OA,** which yields interacting protein-pairs (16, 16) and (16, 1076), and non-interacting protein-pairs (10, 16), (16, 136) and (16, 949). These protein-pairs produce a score of $\log_2(2 / (2 + 3))$. But the maximum score for (Pkinase, Pkinase) under **OA,** denoted as $CB_{\mathbf{OA}}$(Pkinase, Pkinase), is $\log_2(1) = 0.0$, which is obtained through c2 × c2. PG is the number of times a score improves. Evidence that the piggy-backing mechanism does help to improve the score of a domain-pair is given by a negative correlation between average promiscuity and score. As the score increases, the average promiscuity decreases. Promiscuity of a domain-pair $(a, b) = [N(a) + N(b)]/2$.

Piggy-backing: $CB_{\mathbf{OA}}$(Pkinase, Pkinase) > $CB_{\mathbf{Oa}}$(Pkinase, Pkinase) because in **OA** (Figure 6)**,** it is possible for Pkinase to appear with other rare domains, e.g.: c2, c3 and c7. In concept c2, protein 16 interacts with itself and produces the maximum score for (Pkinase, Pkinase). This is an instance of a promiscuous domain (Pkinase) riding piggy-back on rare domains (the Pfam-B domains) to boost the score for (Pkinase, Pkinase). In **Oa**, there is no opportunity for Pkinase to piggy-back on other domains because attribute-labels are reduced. $CB_{\mathbf{Oa}}$(Pkinase, Pkinase) is actually AM(Pkinase, Pkinase), the score obtained via the Associative method. Thus, when the concept lattice is not attribute-reduced, there are more than one context (in the form of different sets of protein-pairs and not in the sense of a formal context in FCA theory) to evaluate the reliability of a domain-pair interaction.

Figure S6 Example of concept-based scoring and piggy-backing with *S. pombe* proteins

Table S2 A cross-table representing the relation between proteins and domains associated with *B. subtilis* in the Riley dataset. E.g.: the domain set for protein 402, D(402) = {HATPase_c, Pfam-B_8931}; and the protein set for domain STAS, P(STAS) = {375, 382, 92}.

		Objects = Proteins (uid)									Domain
		319	375	382	402	409	410	6102	6103	92	Freq.
Attributes = Domains	HATPase_c	x			x						2
	HTH_3							x			1
	Mn_catalase					x					1
	Pfam-B_21839						x				1
	Pfam-B_3091	x									1
	Pfam-B_32775							x			1
	Pfam-B_8931				x						1
	Pfam-B_92151								x		1
	STAS		x	x						x	3
	SpoIIE						x				1
Domains per protein		2	1	1	2	1	2	2	1	1	

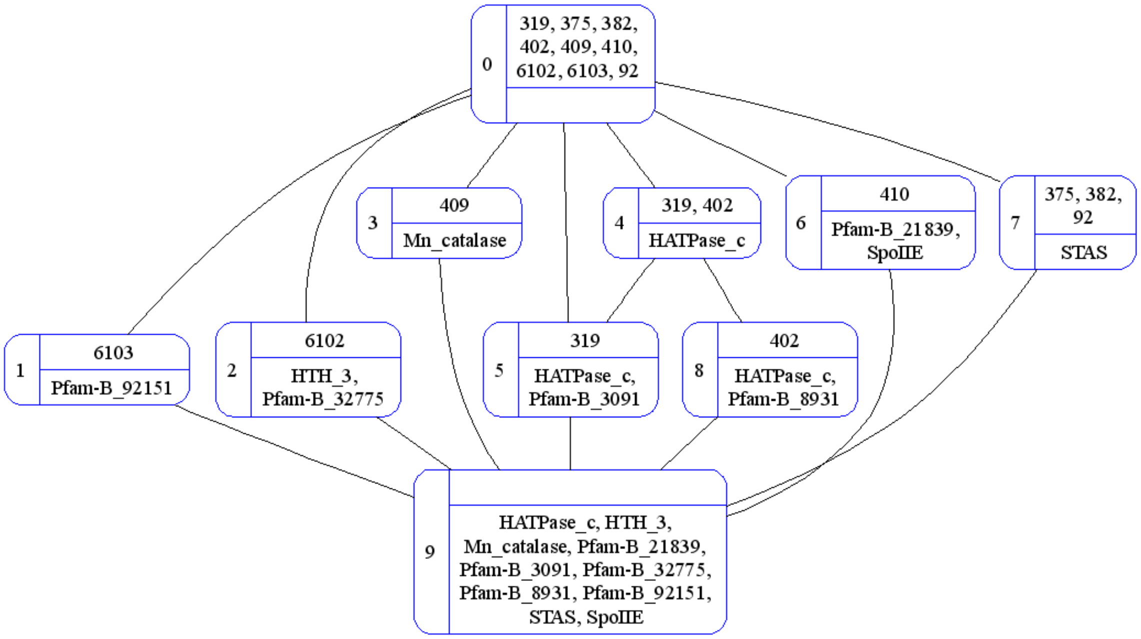

Figure S7 The **OA** concept lattice for the *B. subtilis* context in Table S2.

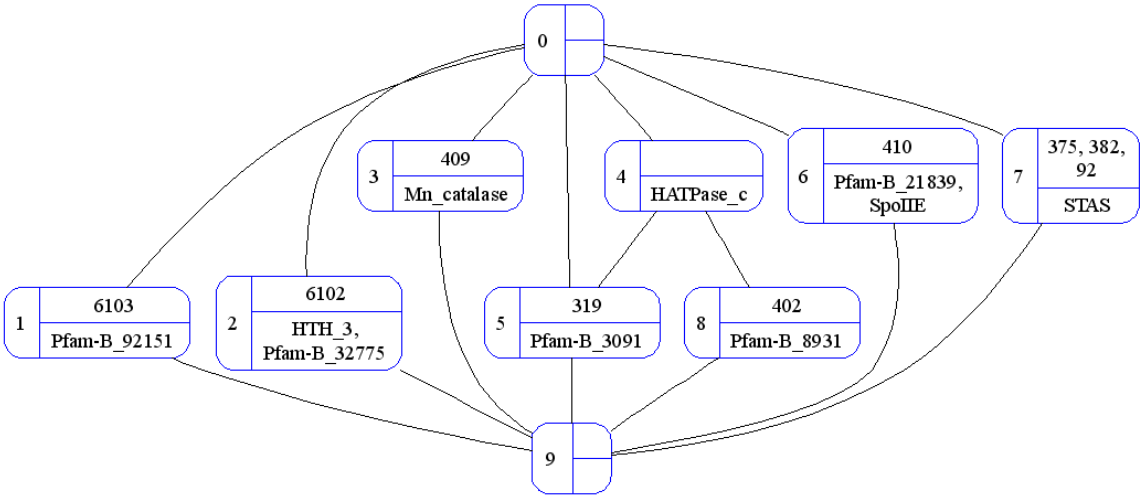

Figure S8 The **oa** concept lattice for the *B.subtilis* context in Table S2.

The nine *B. subtilis* proteins in the Riley dataset. The presence (absence) of an edge between two nodes denotes an interaction (non-interaction). A bolded edge signifies that the two endpoint proteins form a GPPI. A GPPI is a protein-protein interaction that is supported by at least one gold standard domain-pair (GDDI).

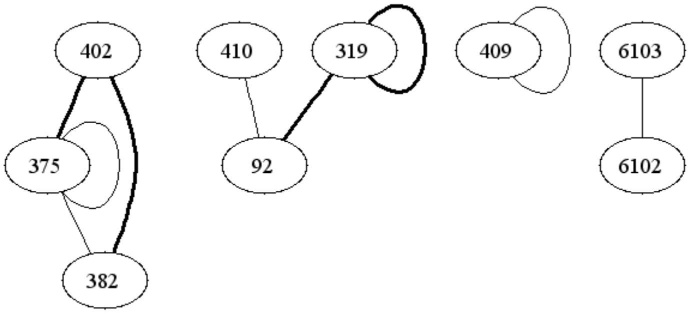

The protein-domain context for *B. subtilis* is in Table S2, and the OA and oa concept lattices are in Figures S7 and S8 respectively.

Concept-based scoring for GDDI(HATPase_c, STAS) with *B. subtilis* PPIs.

Average promiscuity of domain-pairs produced by $A^L(c_1) \times A^L(c_2)$	Concept-pairs (c_1, c_2) for GDDI (HATPase_c, STAS)	**OA**		**oA**		**Oa**		**oa**	
		M	M+Z	M	M+Z	M	M+Z	M	M+Z
2.50	7 × 4	3	6	-	-	**3**	**6**	0	0
2.25	7 × 5	1	3	1	3	-	-	-	-
2.25	7 × 8	**2**	**3**	**2**	**3**	-	-	-	-
Concept-based score CB = max[$\log_2(M/(M+Z))$]		$\log_2 (2/3)$		$\log_2 (2/3)$		$\log_2 (3/6)$		scoreless	
Number of score improvements (piggy-backs) PG		2		1		0		-1	

CB_{OA}(STAS, HATPase_c) > CB_{Oa}(STAS, HATPase_c) because in **OA**, HATPase_c appears with a rare domain (Pfam-B_8931) in c8. In **Oa**, HATPase_c appears only once by definition. As such, there is no opportunity to improve the score of (STAS, HATPase_c). However, it is not sufficient for HATPase_c to appear with a rare domain, it does this also in c5 (Figure S7). CB_{OA}(STAS, HATPase_c) has the score it does because two thirds of the protein-pairs in $O^L(c7) \times O^L(c8)$ are interacting. Hence, the piggy-back of HATPase_c on Pfam-B_8931 in the **OA** concept lattice enables (STAS, HATPase_c) to have the same maximum score as a less promiscuous domain-pair (STAS, Pfam-B_8931). The promiscuity of domain-pair (STAS, HATPase_c) is (3+2)/2=2.5, which is greater than the promiscuity of domain-pair (STAS, Pfam-B_8931) is (3+1)/2=2.0. But CB_{OA}(STAS, HATPase_c) = CB_{OA}(STAS, Pfam-B_8931).

Figure S9 Example of concept-based scoring and piggy-backing with *B. subtilis* proteins.

Table S3 Summary statistics for tests done with the Riley dataset.

Concept lattice type	Scenario	PPIs	DDIs	scored DDIs	GDDIs	scored GDDIs	GDDI/DDI	GPPIs
**oa**	**A**	26032	177233	49,378<br>27.86%	783	350/783<br>44.70%	783/177233<br>0.44%	2326
	**B**	13100	96641	25,693<br>26.59%	587	206/587<br>35.09%	574.25/95901.5*<br>0.60%	1209
	**C**	26032	172291	44,998<br>26.12%	366	153/366<br>41.80%	366/172291<br>0.21%	617
	**D**	26032	194752	28,457<br>14.61%	214	59/214<br>27.57%	214/194752<br>0.11%	1015
**oA**	**A**	26032	177233	100%	783	100%	0.44%	2326
	**B**	13013	93820	100%	570	100%	0.60%	1179
	**C**	26032	172291	100%	366	100%	0.21%	617
	**D**	26032	194752	100%	214	100%	0.11%	1015
**Oa**	**A**	26032	177233	100%	783	100%	0.44%	2326
	**B**	13007	97206	100%	569	100%	0.60%	1137
	**C**	26032	172291	100%	366	100%	0.21%	617
	**D**	26032	194752	100%	214	100%	0.11%	1015
**OA**	**A**	26032	177233	100%	783	100%	0.44%	2326
	**B**	12936	95939	100%	571	100%	0.60%	1176
	**C**	26032	172291	100%	366	100%	0.21%	617
	**D**	26032	194752	100%	214	100%	0.11%	1015

* 574.25 = (587 + 570 + 569 + 571) /4; 95901.5 = (96641 + 93820 + 97206 + 95939)/4

Table S4 Number of concepts used for scoring domain-pairs. Only concepts where both object-label and attribute-label sets are not empty are used. The total number of concepts (including the top and bottom concepts) generated for the Riley dataset is 8,894. This number increases to 11,034 when the domains are shuffled, which indicates a more fragmented protein domain context. In scenario **D**, rare and promiscuous domains are no longer "glued" together by mixed architecture proteins.

Concept lattice type	Original (scenarios **A**, **B** and **C**)	Domain shuffled (scenario **D**)
**oa**	6,564	943
**oA**	7,325	5,926
**Oa**	7,639	5,917
**OA**	8,892	11,032

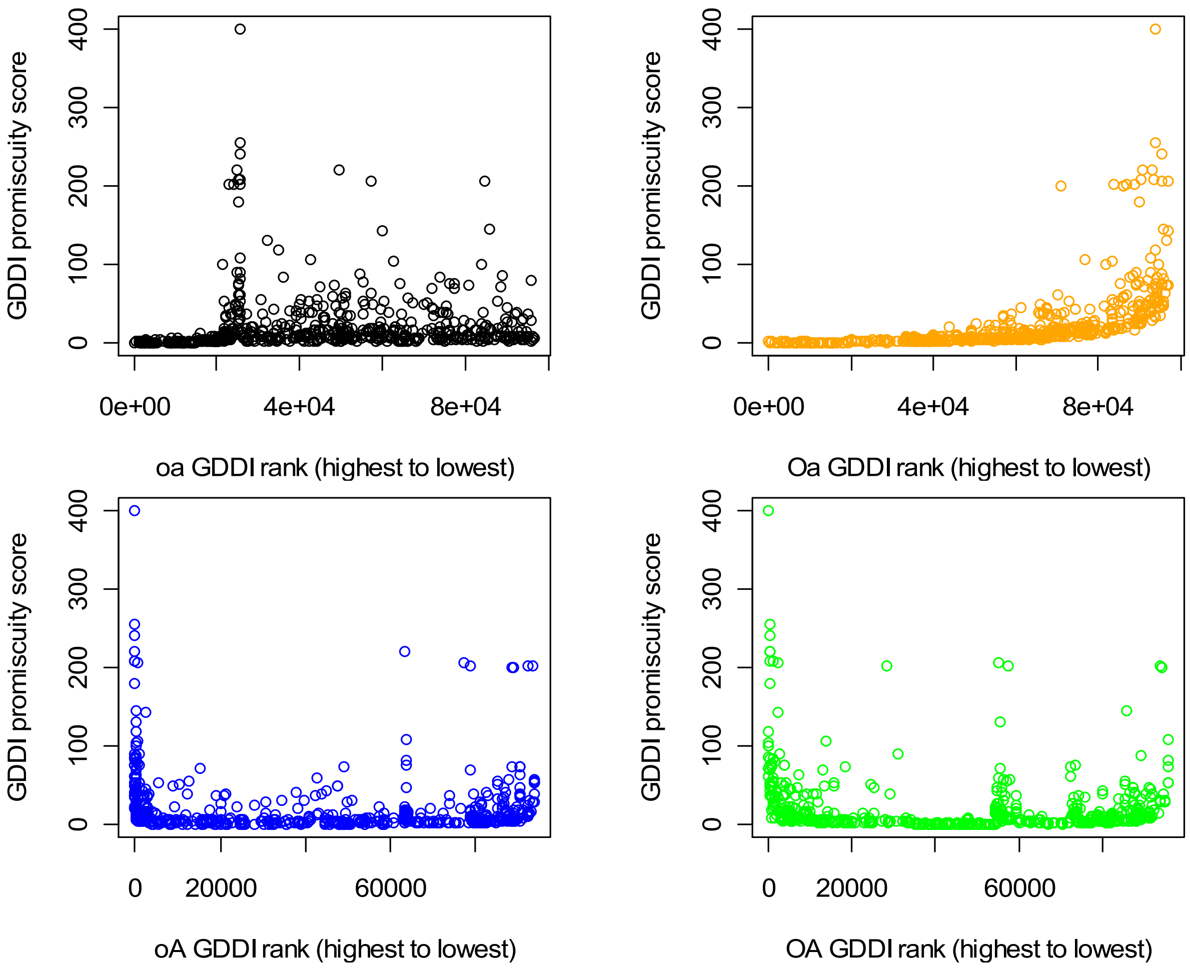

Figure S10 Scatter-plot of GDDI rank vs. promiscuity, scenario B Pe=0.5. Reducing PPIs did not qualitatively change the relationship between GDDI rank and promiscuity. All the putative DDIs were ranked as described in the text, and the ranks of GDDIs were extracted to create the plots. Promiscuity of a domain pair (*a*, *b*) = [N(*a*) + N(*b*)]/2 where N(*d*) is the number of times domain *d* occurs in a protein set. The **OA** and **oA** concept lattices still exhibit the desired negative relationship, which means in spite of the reduction in PPIs, the non-attribute-reduced concept lattices still tend to rank promiscuous GDDIs more highly. The relationship is still strongly positive when the **Oa** rankings are used. **Oa** results are identical to the Associative method which is known to penalize promiscuous domain-pairs. There is still also a tendency for the **oa** concept lattice to rank promiscuous GDDIs less highly, but this positive relationship is not so apparent because scoreless GDDIs are included in the plot (they start at rank 25,693 and onwards to the right of the plot). When the **oa** concept lattice is used, only 206 of the 587 GDDIs have CB scores; the remaining GDDIs are scoreless and are ranked randomly but below the GDDIs with CB scores.

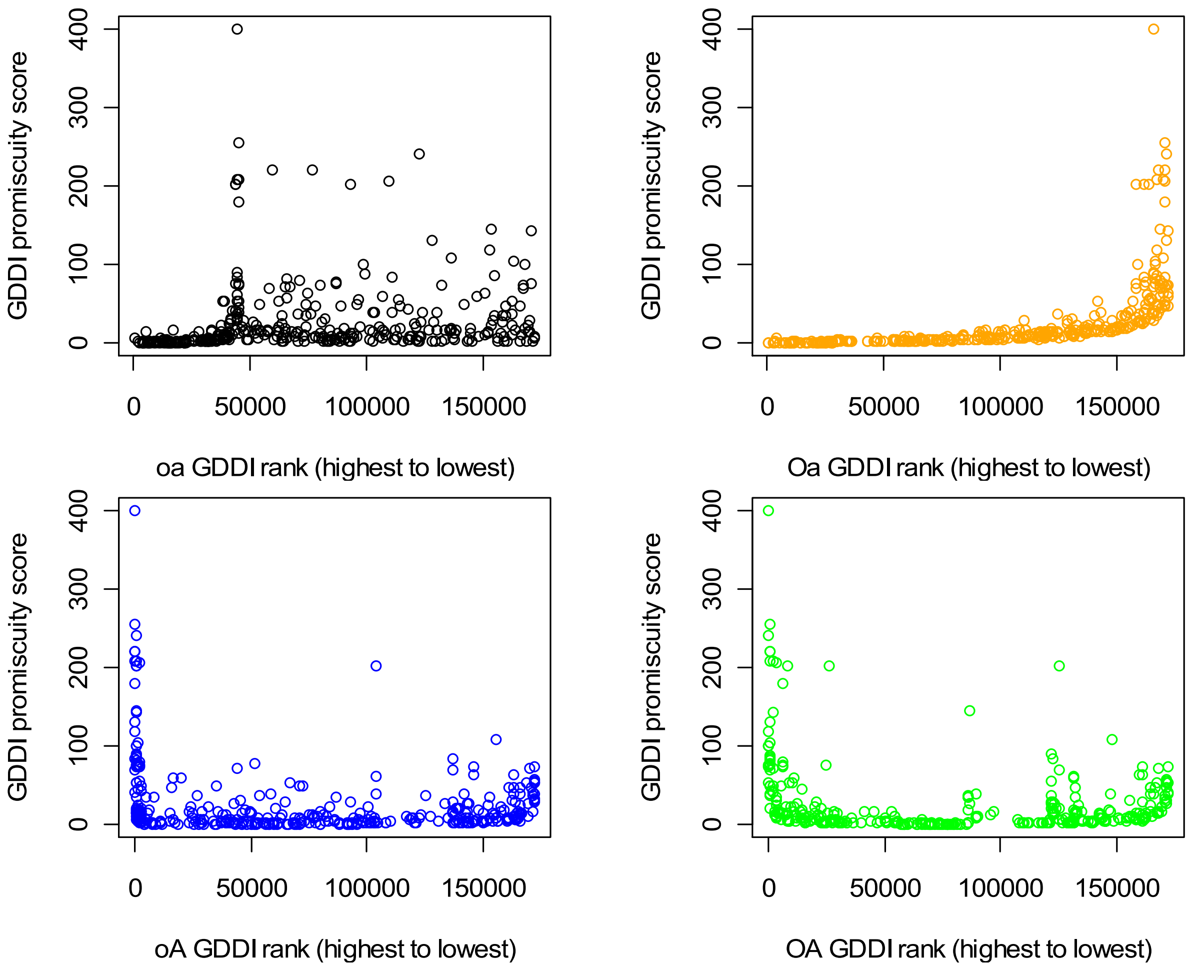

Figure S11 Scatter-plot of GDDI rank vs. promiscuity, scenario C shuffled proteins, Pe=1.0. Changing the PPIs did not qualitatively change the relationship between GDDI rank and promiscuity. All the putative DDIs were ranked as described in the text, and the ranks of GDDIs were extracted to create the plots. Promiscuity of a domain pair (a, b) = [N(a) + N(b)]/2 where N(d) is the number of times domain d occurs in a protein set. The **OA** and **oA** concept lattices still exhibit the desired negative relationship, which means in spite of the changes to the PPIs, the non-attribute-reduced concept lattices still tend to rank promiscuous GDDIs more highly. The relationship is still strongly positive when the **Oa** rankings are used. **Oa** results are identical to the Associative method which is known to penalize promiscuous domain-pairs. There is still also a tendency for the **oa** concept lattice to rank promiscuous GDDIs less highly, but this positive relationship is not so apparent because scoreless GDDIs are included in the plot (they start at rank 44,998 and onwards to the right of the plot). When the **oa** concept lattice is used, only 153 of the 366 GDDIs have CB scores; the remaining GDDIs are scoreless and are ranked randomly but below the GDDIs with CB scores.

The *S. pombe* protein-pair (16, 1076) is a GPPI since it is supported by the (Pkinase, Pkinase) GDDI. This protein-pair generates the four DDIs listed in the table below. The GDDI (Pkinase, Pkinase) is the highest ranking DDI only when the **OA** concept lattice is used. Because its object-labels are not reduced, **OA** concept lattices generate a larger range of PG values and for more domain-pairs than **oA** (Figure 14). This explains the better Nye test performance of the **OA** concept lattice over the **oA** concept lattice (Figure 18). PG values help differentiate domain-pairs with identical CB values. PG ≤ 0 for attribute-reduced concept lattices since attribute-labels appear only once and thus there is no chance for piggy-backing. However, it is possible to go overboard with the piggy-backing and for a few GPPIs (Figure 19).

DDIs for GPPI(16, 1076)	**OA**		**oA**		**Oa**		**oa**	
	**CB**	**PG**	**CB**	**PG**	**CB**	**PG**	**CB**	**PG**
(Pkinase, Pfam-B_106217)	0	1	0	1	-1.32193	0	-1	0
(Pkinase, Pfam-B_33993)	0	1	0	1	-1.32193	0	-1	0
(Pkinase, Pfam-B_78326)	0	1	0	1	-1.32193	0	-1	0
(Pkinase, Pkinase)	0	2	0	1	-2.90689	0	scoreless	-1
GDDI (Pkinase, Pkinase) is the highest ranked DDI?	Yes		No		No		No	

The three GPPIs for *S. pombe* in the Riley dataset are listed in the table below. Only rankings made with the **OA** concept lattice correctly placed a GDDI as the highest ranking DDI for all three GPPIs. The other three concept lattice types correctly placed a GDDI as the highest ranking DDI for only one of the GPPIs. But this GPPI has a GDDI/DDI ratio of 1.0 which is hardly a challenge for the Nye test.

GPPI	Number of DDIs	GDDI	**OA**	**oA**	**Oa**	**oa**
(319, 319)	3	(HATPase_c, HATPase_c)	Yes	No	No	No
(319, 92)	2	(STAS, HATPase_c)	Yes	Yes	Yes	No
(375, 402)	2	(STAS, HATPase_c)	Yes	Yes	No	No
(382, 402)	2	(STAS, HATPase_c)	Yes	Yes	No	No
Number of GPPIs with a GDDI as the highest ranking DDI			4	3	1	0

Figure S12 An application of the Nye test.

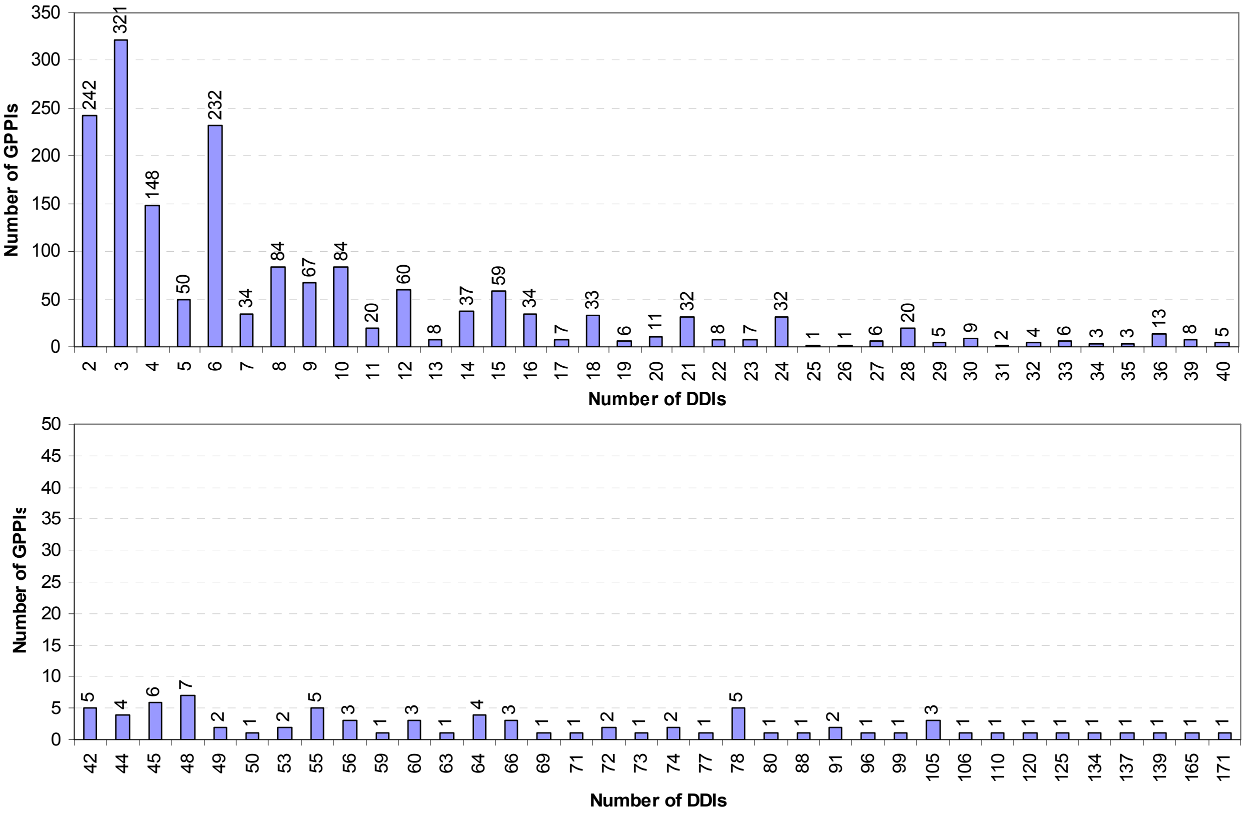

Figure S13 Number of GPPIs that generate *x* number of DDIs.

Table S5 Summary statistics for tests done with the Biogrid-PfamA-3did dataset

	Yeast		Human	
Proteins (mapped to Uniprot ID)	4,872		22,821	
Pfam-A domains	2,933		5,456	
Biogrid PPIs "Physical association"	50,170		72,396	
DDIs	71,213		110,240	
3did DDIs	1,295		1,509	
Concepts				
**oa**	2,482	30,757 scoreless domain-pairs	4,723	79,107 scoreless domain-pairs
**oA**	2,666	No scoreless domain-pairs	6,796	No scoreless domain-pairs
**Oa**	2,572		4,985	
**OA**	2,757		7,349	
